# Enantioselective and diastereoselective azo-coupling/iminium-cyclizations: a unified strategy for the total syntheses of (–)-psychotriasine and (+)-pestalazine B†
†Electronic supplementary information (ESI) available: Experimental, characterization data, X-ray structures of compound **15**, and NMR spectra. CCDC 1040494. For ESI and crystallographic data in CIF or other electronic format see DOI: 10.1039/c5sc00338e


**DOI:** 10.1039/c5sc00338e

**Published:** 2015-05-05

**Authors:** Qi Li, Tingting Xia, Licheng Yao, Haiteng Deng, Xuebin Liao

**Affiliations:** a Tsinghua-Peking Centre for Life Sciences , Beijing 100084 , China; b Department of Pharmacology and Pharmaceutical Sciences , School of Medicine , Collaborative Innovation Center for Diagnosis and Treatment of Infectious Diseases , Tsinghua University , Beijing 100084 , China . Email: liaoxuebin@mail.tsinghua.edu.cn; c MOE Key Laboratory of Bioinformatics , School of Life Sciences , Tsinghua University , Beijing 100084 , China . Email: dht@mail.tsinghua.edu.cn

## Abstract

We report a unified strategy for the total syntheses of (–)-psychotriasine and (+)-pestalazine B based on the advanced intermediates of 3α-amino-hexahydropyrrolo[2,3-*b*]indole. To construct these structural motifs, a cascade reaction involving a BINOL-derived phosphoric anion-paired catalyst for enantioselective or diastereoselective azo-coupling/iminium-cyclizations was developed. The remaining key steps of the synthesis involve a sterically hindered amination *via* hypervalent iodine reagents and the Larock annulation. These transformations enable a general approach to the syntheses of indole alkaloids containing a 3α-amino-hexahydropyrrolo[2,3-*b*]indole motif and could be further applied to build a natural product-based library.

## Abstract 2

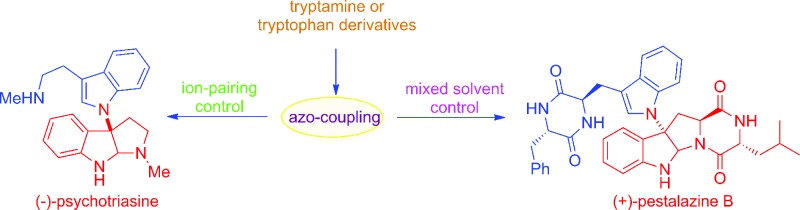
We report a unified strategy for the total syntheses of (–)-psychotriasine and (+)-pestalazine B based on the advanced intermediates of 3α-amino-hexahydropyrrolo[2,3-*b*]indole.

## Introduction

**Fig. 1:**
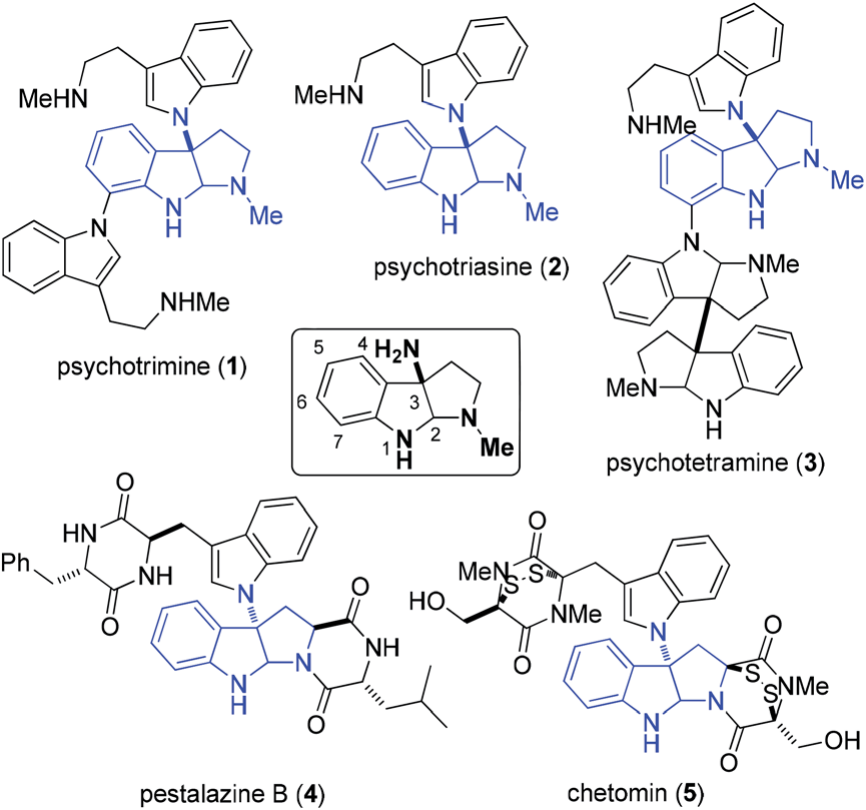
Compounds containing the 3α-amino-hexahydropyrrolo[2,3-*b*]indole core.

Indole alkaloids containing a 3α-amino-hexahydropyrrolo-[2,3-*b*]indole scaffold are present in a wide range of natural compounds (Fig. 1).^1^ Structurally, these indole alkaloids feature the linkage of two or more tryptamines or tryptamine derivatives *via* C3a–N1 bond formation. Due to their unique structures and versatile biological activities these compounds have attracted significant interest from synthetic chemists. For example, psychotrimine (**1**) was first isolated by the Takayama group and later synthesized by Takayama, Baran and other groups.^2^,^3^ It exhibits antibacterial activity against Gram-positive bacteria through membrane disruption.^4^ Chetomin (**5**) is a potential anti-cancer agent against the transcription factor hypoxia inducible factor 1 (HIF-1).^5^ Williams and co-workers have made great progress in the biosynthesis of chetomin.5*e* Psychotetramine (**3**) was the first reported alkaloid bearing C3–C3, C7–N1 and C3a–N1 linkages. It was isolated by the Takayama group and its structure was recently elucidated by Baran *et al.*^6^ Among other alkaloids in this family, psychotriasine (**2**) was isolated by the Hao group,^7^ while pestalazine B (**4**) was isolated by the Che group and its total synthesis and structural revision was completed by de Lera and co-workers.^8^

**Fig. 2:**
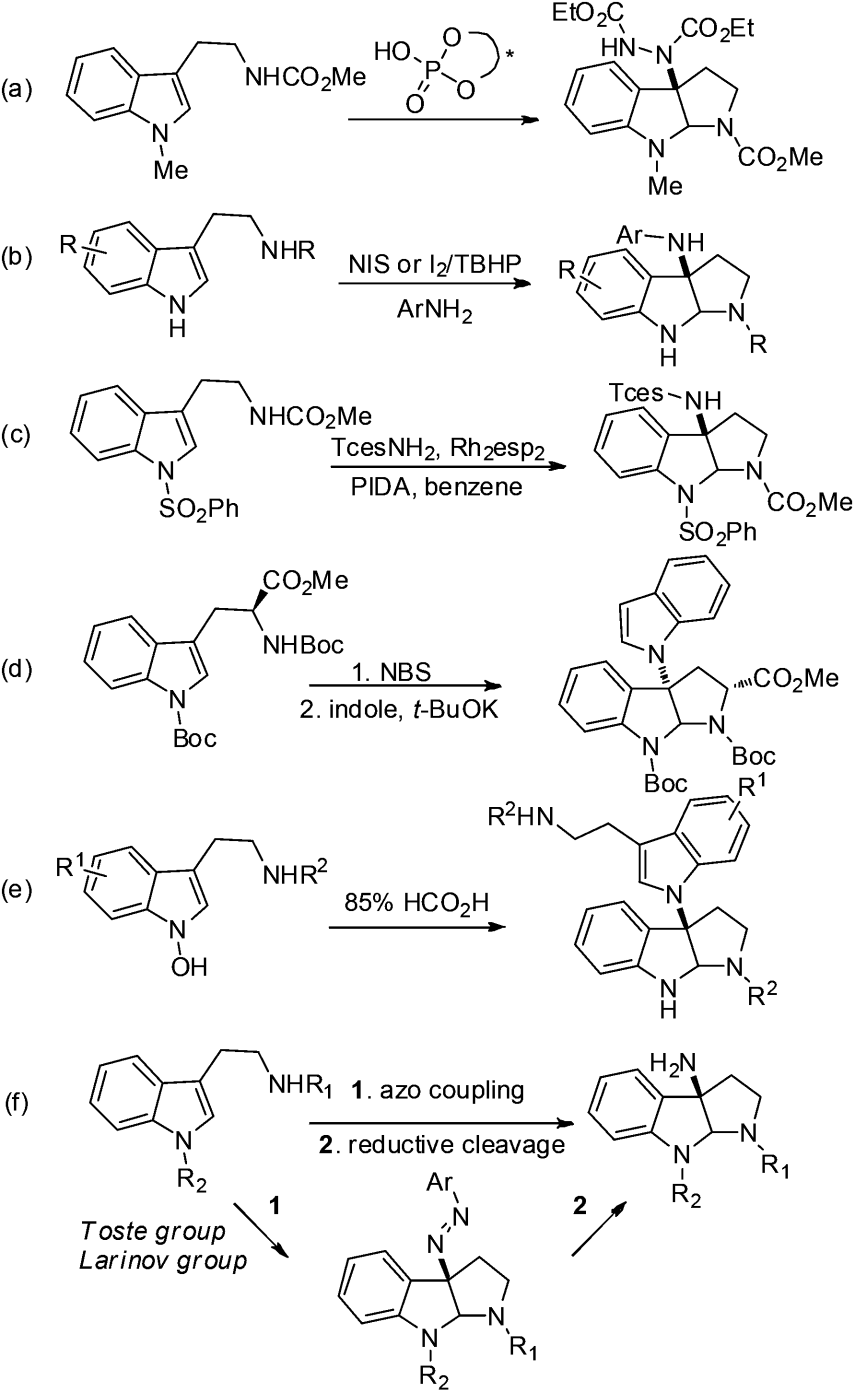
Reported synthetic methods for the 3α-amino-hexahydropyrrolo[2,3-*b*]indole core structure (a–e) and our approach in this publication and others^14^ (f).

To develop a unified strategy for the total synthesis of these C–N linked indole alkaloids, we needed to establish a highly efficient method for rapid construction of the 3α-amino-hexahydropyrrolo[2,3-*b*]indole core structure. To date, a number of synthetic approaches to this core structure have been developed and are summarized in Fig. 2: (a) a well-designed method that involves a BINOL-derived chiral phosphoric acid catalyzing the addition of tryptamine derivatives with DEAD was achieved by Antilla and his colleagues;^9^ (b) an elegant approach to form a C–N bond through coupling of tryptamine derivatives with aniline in the presence of appropriate oxidants was accomplished independently by the Baran and Ji groups;3b,d,^6^,^10^ (c) a nitrene-induced aziridination followed by a ring-opening and imine cyclization cascade reaction was developed by Dauban and co-workers, which readily afforded 3α-amino-hexahydropyrrolo[2,3-*b*]indole derivatives;^11^ (d) a recent report by the Rainer group on an expeditious synthesis of 3α-indole-hexahydropyrrolo[2,3-*b*]indole *via* the cyclopropane ring-opening process was disclosed;^12^ (e) an early report on an acid-catalyzed nucleophilic substitution reaction on the indole nitrogen was described by the Somei group.^13^

Most recently, hinging on their signature work on phase transfer catalysis, the Toste group reported a highly enantioselective (up to 96% ee) reaction to construct C3-diazenated pyrroloindolines *via* a chiral anion phase transfer process (Fig. 2f).14*a* This elegant work once again demonstrated the beauty of the chiral anion pair concept in asymmetric catalysis. Shortly after, the Larionov group disclosed a racemic version of this reaction.14*b* However, this capable method to construct the 3α-amino-hexahydropyrrolo[2,3-*b*]indole scaffold was not available when we started the current investigation of our total syntheses. Thus, we have independently pursued the strategy of stereoselective domino azo-coupling/iminium cyclizations with N_a_–H tryptomine derivatives as a key step for our total synthesis design. In particular, our methodology development includes an enantioselective azo-coupling catalysis that was very similar to the Toste group's approach, as well as a new process of solvent-enabled diastereo-selective azo-coupling. Both methods allowed us to readily prepare C3-diazenated pyrroloindoline structural motifs *via* a cascade process. This intermediate could then be subjected to reducing conditions, and the desired 3α-aminohexahydro-pyrrolo[2,3-*b*]indole scaffold would be synthesized. Herein we report the successful application of these stereoselective cascade processes in the total syntheses of (–)-psychotriasine (**2**) and (+)-pestalazine B (**4**). These results demonstrate their utility as a unified strategy to synthesize such indole alkaloids.

## Results and discussion

Diazonium salts can be generally utilized as nitrogen sources in organic synthesis. A prominent example of their synthetic application was described in Fukuyama's total synthesis of mersicarpine.^15^ Similar to Toste's study, we envisioned that the chiral ion pair^16^–^18^ which was generated by mixing diazonium salts, Na_2_CO_3_ and chiral phosphoric acid,^19^ would couple with tryptamine derivatives to form iminium intermediates. We anticipated that iminium cyclization would then occur to yield the azo compound in an enantioselective or diastereoselective fashion, which was the precursor of 3α-amino-hexahydropyrrolo[2,3-*b*]indole (Fig. 2f).

**Scheme 1:**
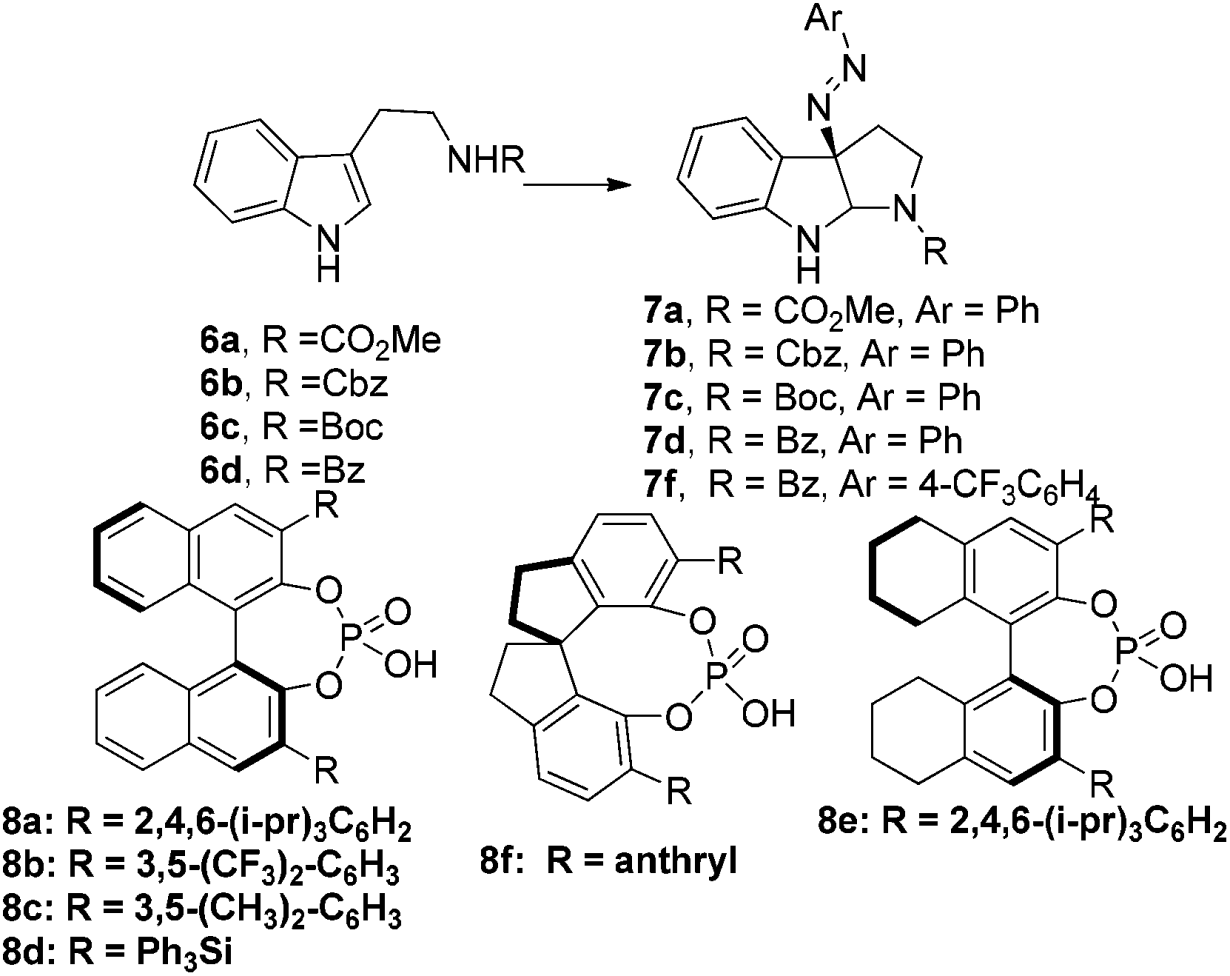
Enantioselective azo-coupling of tryptamine derivatives.

We explored the above strategy using N_a_–H tryptamine derivative **6a** (R = CO_2_Me) and PhN_2_BF_4_ as the model substrates (Scheme 1). Initially (*S*)-TRIP^20^ was used as the chiral phosphoric acid catalyst (**CPA8a**) at 0 °C in different solvents (for details see ESI, S-Table 1†). We were encouraged by the formation of desired product **7a** in good yields and moderate er (up to 67 : 33) in ethereal solvents such as *t*-BuOMe. Those results demonstrated that it was possible to construct the target core structure in high enantioselectivity by the chiral ion pair strategy. When switching the N-protecting group from CO_2_Me to Cbz, Boc or Bz, we found that the Bz-protected product **7d** was formed with the highest enantioselectivity (27 : 73 er). Further screening of the reaction parameters showed a slight increase in enantioselectivity at a lower temperature of –40 °C (25 : 75 er), while using other CPA (**8b–e**) catalysts led to similar or lower enantioselectivity. To our surprise, when we used 0.01 equiv. of pyridine as an additive, the enantioselectivity was increased to 20 : 80 er (CPA: **8a**). Replacing pyridine with lutidine gave similar results, and using 2,6-di-*t*-butylpyridine led to further improvement of enantioselectivity to 14.5 : 85.5 er. Presumably, a more compact and lipophilic ion pair may result in a higher enantioselectivity, and pyridine-type additives may be involved in the formation of tight ion pairs. Further optimization of the reaction conditions was achieved by applying 4-CF_3_-PhN_2_BF_4_ as the diazonium salt instead of PhN_2_BF_4_ and lowering the reaction temperature to –60 °C to obtain product **7f** in quantitative yield and 7.5 : 92.5 er (CPA: **8a**). Additional purification of **7f** by recrystallization in petroleum ether led to 69% yield and >96% ee. Our findings, in retrospection, are in good agreement with the results of the Toste group.

**Scheme 2:**
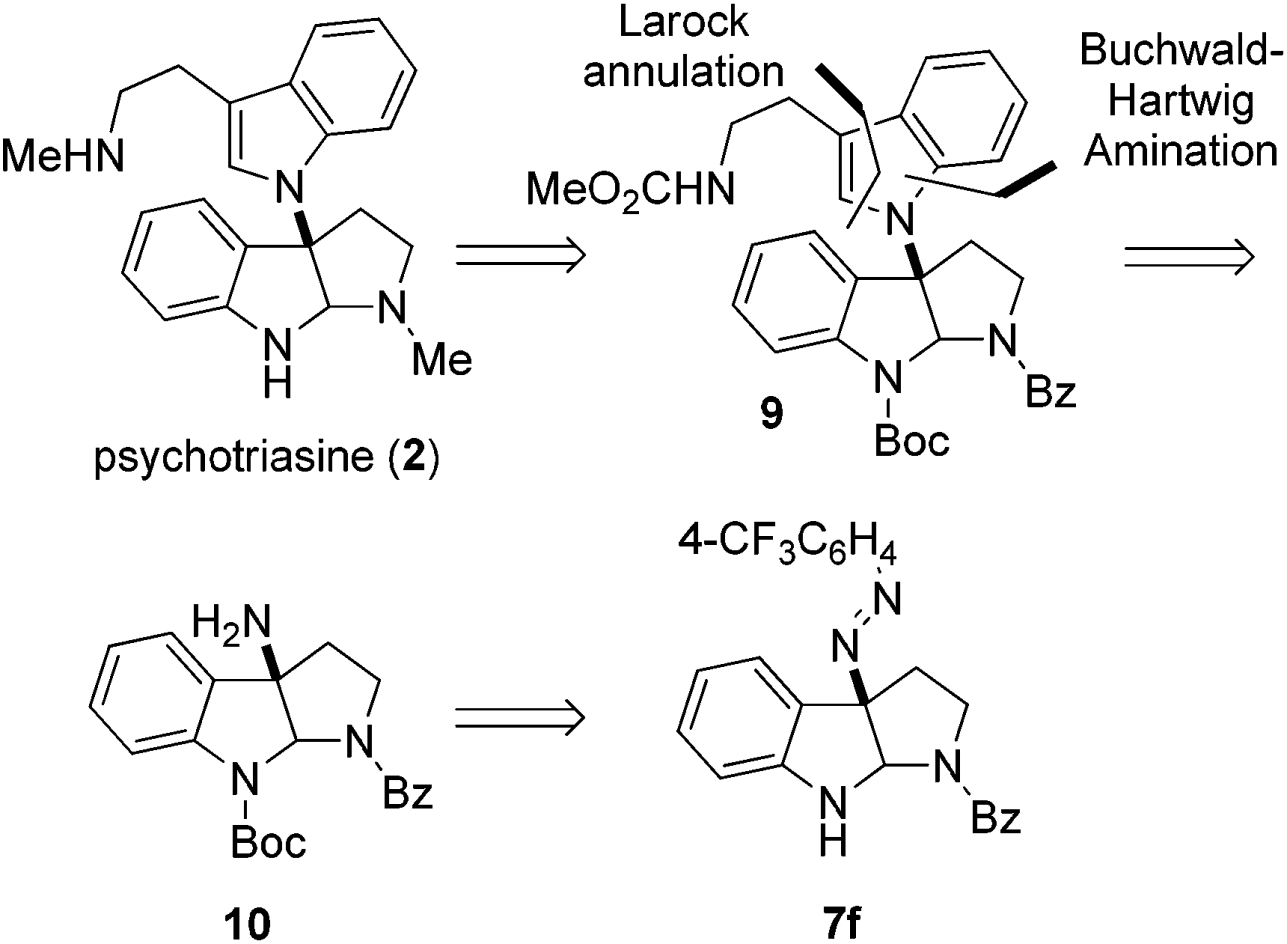
Retrosynthetic analysis of (–)-psychotriasine (**2**).

With the core structure of 3α-amino-hexahydropyrrolo[2,3-*b*]indole **7f** obtained in high ee, we strived to complete the enantioselective total synthesis of natural product (–)-psychotriasine (**2**). Our retrosynthetic analysis is illustrated in Scheme 2; we proposed that (–)-psychotriasine could be readily achieved *via* several functional transformations from N_a_-Boc protected compound **9**. Compound **9** could be constructed by a Pd-catalyzed Buchwald–Hartwig amination followed by Larock annulation from the amino compound **10**. Lastly, amine **10** could be synthesized from compound **7f** through a few simple manipulations.

**Scheme 3:**
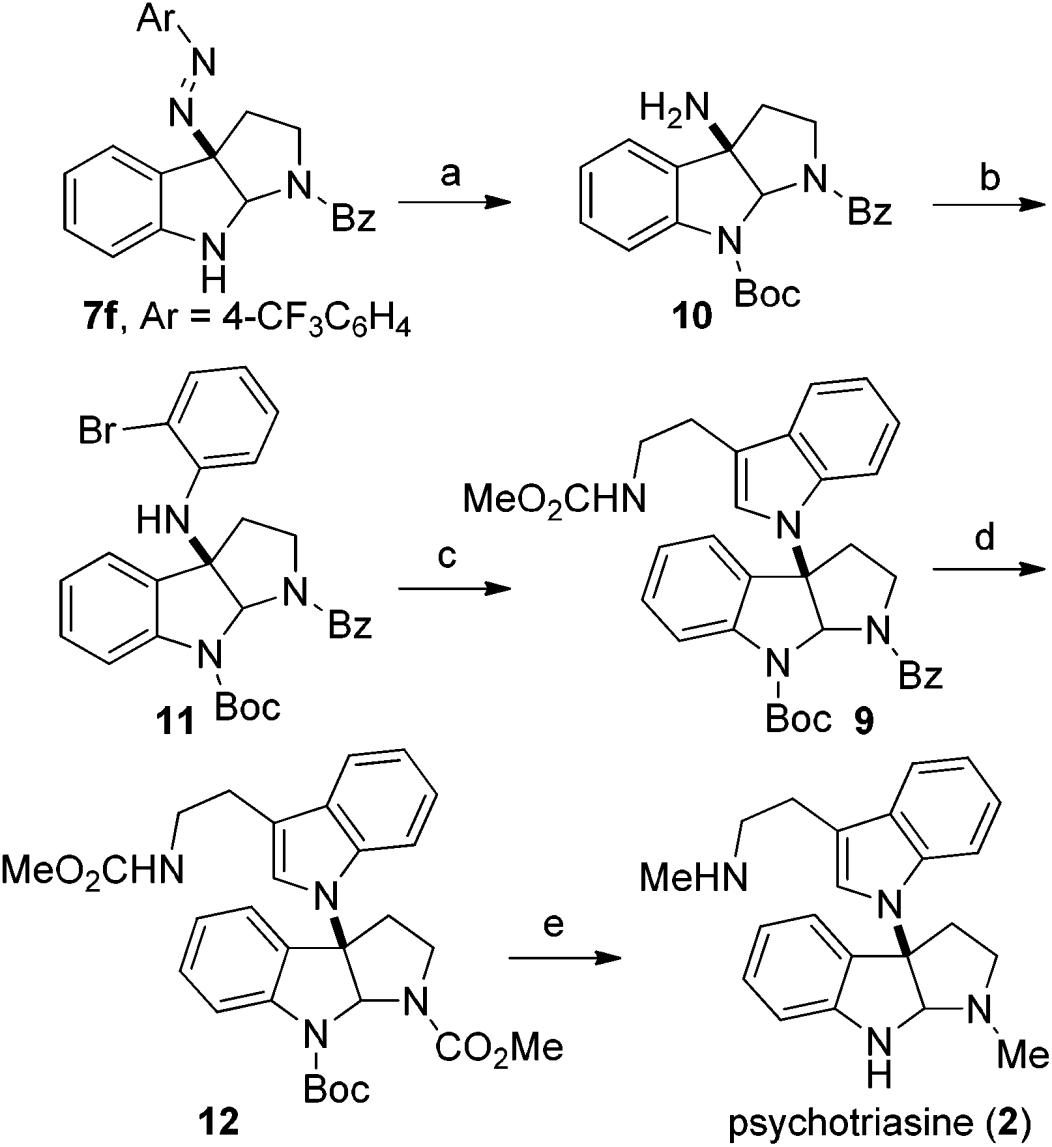
Total synthesis of (–)-psychotriasine (**2**). Reagents and conditions: (a) (i) NaHMDS, Boc_2_O, THF, –60 °C; (ii) RANEY® nickel, hydrazine hydrate, MeOH, sealed tube, 70 °C, 88% yield over 2 steps; (b). Pd(OAc)_2_ (0.2 equiv.), xantphos (0.3 equiv.), *t*-BuONa (2.0 equiv.), toluene, 80 °C, 71% yield; (c) **13**, Pd(OAc)_2_ (0.2 equiv.), D^*t*^BPF (0.3 equiv.), K_2_CO_3_ (2.5 equiv.), NMP, 110 °C, 83% yield; (d) (i) DIBAL-H (3.0 equiv.), toluene, –78 °C; (ii). ClCO_2_Me (3.0 equiv.), Na_2_CO_3_ (3.0 equiv.), DCM–H_2_O (2 : 1 ratio), rt, 64% yield over 2 steps; (e) (i) CF_3_CO_2_H, DCM, 0 °C; (ii) Red-Al (12.0 equiv.), toluene, reflux, 51% yield over 2 steps. Alkyne **13**: *N*-(methoxycarbonyl)-4-(trimethylsilyl)-3-butynylamine.

Based on the retrosynthetic analysis, our total synthesis of (–)-psychotriasine began with **7f** which was readily accomplished using our newly developed method (Scheme 3). The indole nitrogen in compound **7f** was first protected with Boc in the presence of NaHMDS and Boc_2_O at –60 °C under argon. However, subsequent cleavage of the *N*,*N* double bond was not trivial, and very poor results were acquired when we tried a variety of reductive cleavage conditions including hydrogenolysis with H_2_ under different pressures and temperatures, and even reduction with TiCl_3_/NaOH. We then attempted using hydrazine hydrate in the presence of RANEY® nickel, and it was discovered that only when excess hydrazine hydrate and a catalytic amount of RANEY® nickel were utilized could the desired product **10** be obtained in a satisfactory yield over 2 steps (88%).^21^,^22^ Compound **10** was coupled with 1,2-dibromobenzene in the presence of a Pd(OAc)_2_ catalyst, xantphos ligand and *t*-BuONa as base additive to afford the Buchwald–Hartwig amination product **11** in 71% yield.^23^ Next, the tryptamine dimer compound **9** was obtained *via* Larock cyclization from **11** and a known alkyne building block (*N*-(methoxycarbonyl)-4-(trimethylsilyl)-3-butynylamine) **13** (ref. 24) using a Pd(OAc)_2_ catalyst, D^*t*^BPF ligand, K_2_CO_3_ additive and NMP solvent in 83% yield.^25^ N-deprotection for Bz removal with DIBAL-H^26^ followed by carbamation of the amine with ClCO_2_Me gave **12** in 64% yield over 2 steps. Finally, N-deprotection of **12** to remove the Boc group with CF_3_CO_2_H and further reduction with Red-Al provided the desired natural product (–)-psychotriasine (**2**) in >96% ee.3*b* With an overall yield of 11.7% over 9 steps, this procedure represents the first enantioselective total synthesis of (–)-psychotriasine.

**Fig. 3:**
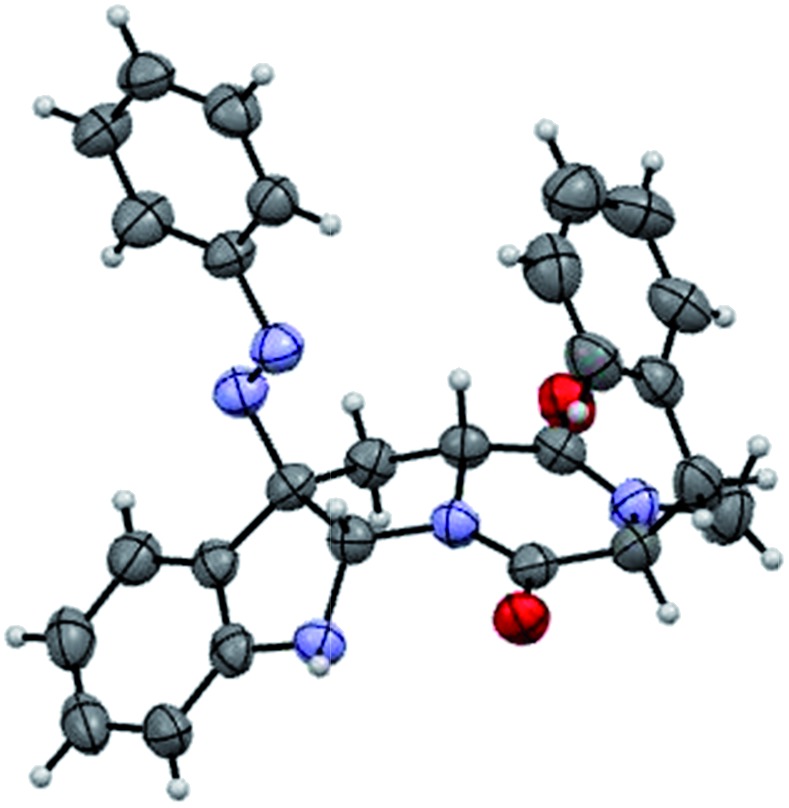
Thermal ellipsoid plot of the X-ray structure of compound **16** at the 50% probability level.

With the successful synthesis of (–)-psychotriasine, we envisioned that other natural products containing the 3α-amino-hexahydropyrrolo[2,3-*b*]indole core structure could also be synthesized *via* enantioselective azo-coupling of simple tryptamine derivatives with diazonium salts. However, analogous natural products bearing an extended core structure with the cyclic dipeptide motif, such as (+)-pestalazine B (**4**) and chetomin (**5**), couldn't be obtained directly. Baran's group reported an elegant approach to form a C–N bond through tryptamine derivatives coupling with aniline in the presence of appropriate oxidants;3d,^6^ however, when this method was attempted with **14**, only a trace amount of the coupling product was obtained. To address this synthetic challenge, we sought to develop a highly diastereoselective azo-coupling of tryptophan derivatives. To obtain better diasteroselectivity of the azo-coupling reaction, we chose compound **14** as a model substrate to screen different solvents and reaction temperatures (Table 1). Using THF as solvent at 15 °C, diastereomer **16** was formed exclusively but only in 14% yield (entry 5). In comparison, utilizing DCE as solvent led to the formation of both diastereomers in a 82% combined yield and high selectivity for **16** (5 : 1, **16** : **15**) (entry 6). Evaluation of a mixed THF–DCE solvent system (entries 8–11) led to an optimized solvent ratio of 1.5 : 1 DCE : THF that promoted a highly diastereoselective azo-coupling to afford **16** in 61% isolated yield and >25 : 1 diastereoselectivity. The configuration of **16** was confirmed by single crystal X-ray diffraction (Fig. 3). With this result in hand, we decided to pursue the total synthesis of (+)-pestalazine B (**4**).

**Table 1:** Diastereoselective azo-coupling of tryptophan derivatives^*a*^

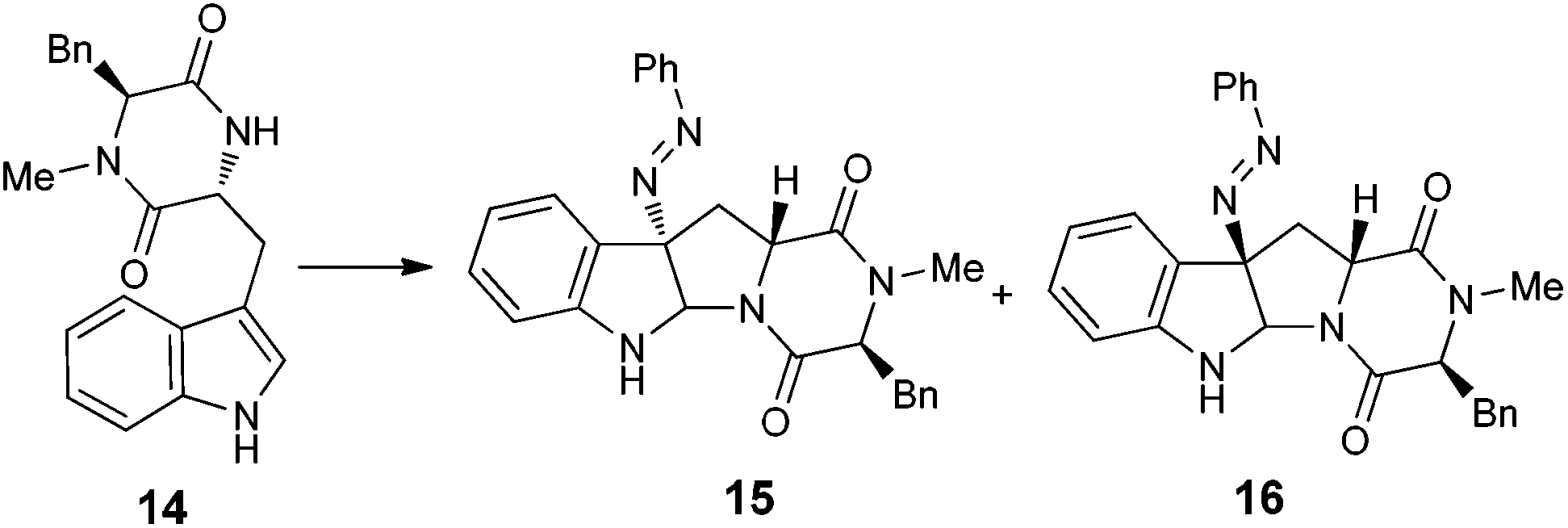					
Entry	Solvent	*T* (°C)	Reaction time	**15** : **16**	Yield^*b*^ (%)
1	MeOH	–78	1 h	1 : 1	66%
2	MeCN	rt	24 h	—	—^*c*^
3	Toluene	rt	24 h	1 : 1.5	41%
4	THF	rt	24 h	1 : 1.5	74%
5	THF	15	24 h	Only **16**	14%
6	DCE	15	24 h	1 : 5	82%
7	DCE	rt	24 h	1 : 5	57%
8	DCE–THF(1 : 1)	15	48 h	1 : 10	63%
9	DCE–THF(1.5 : 1)	15	48 h	<1 : 25	67% (61^*d*^%)
10	DCE–THF(2 : 1)	15	96 h	1 : 20	50%
11	DCE–THF(4 : 1)	15	96 h	1 : 4	43%

^*a*^Conditions: **14** (0.1 mmol), PhN_2_BF_4_ (0.11 mmol), K_2_CO_3_ (0.15 mmol) and solvent (1 mL).

^*b*^NMR yield with 1,3,5-trimethoxylbenzene as internal standard.

^*c*^No desired product was detected.

^*d*^Isolated yield.

**Scheme 4:**
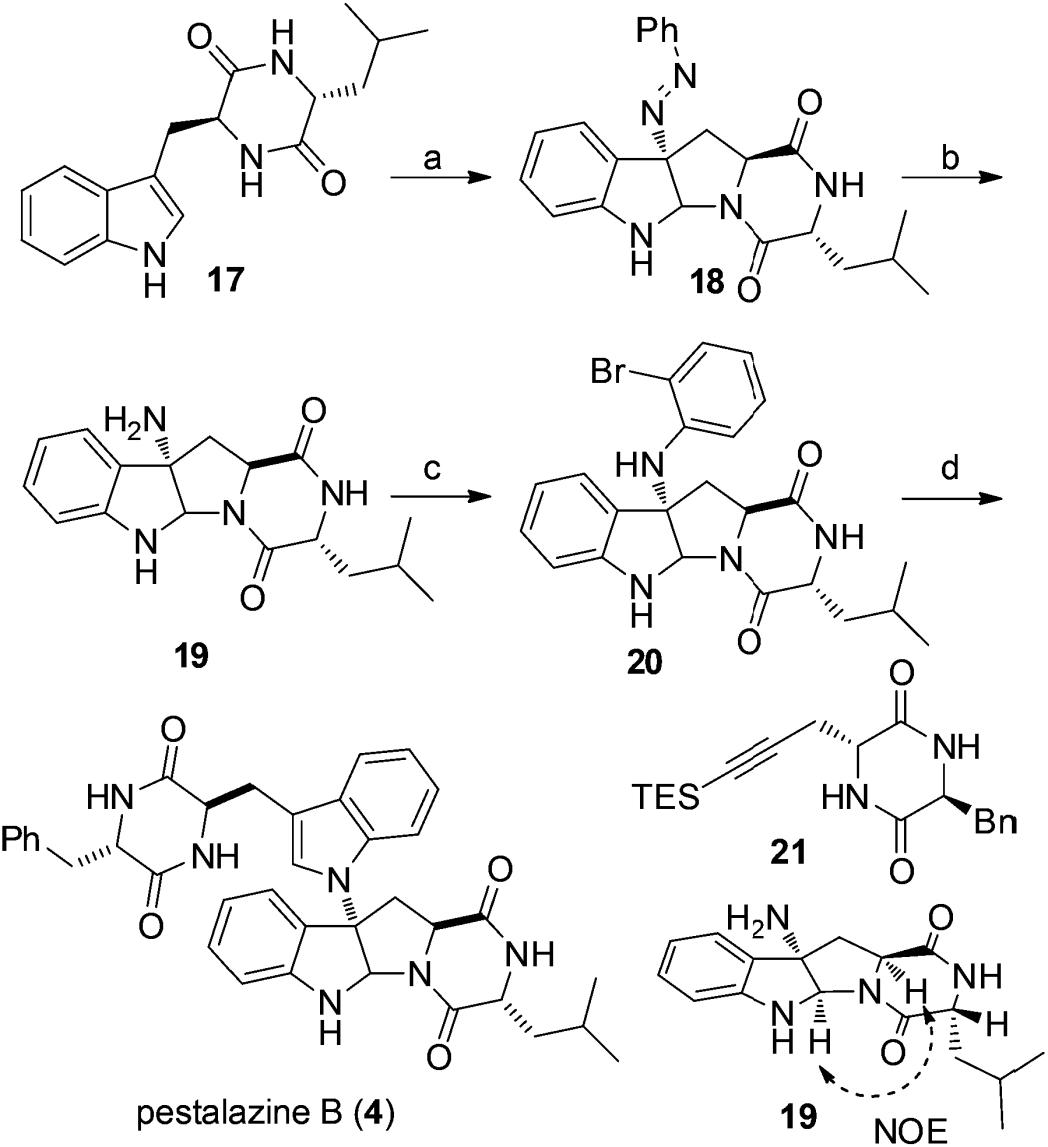
Total synthesis of (+)-pestalazine B (**4**). Reagents and conditions: (a) PhN_2_BF_4_, K_2_CO_3_, **CPA8a**, DCE–THF (1.5 : 1), 0 °C, 79% yield; (b) hydrazine hydrate, Pd/C, EtOH, 85 °C, 80% yield; (c) **A3**, Cu(OTf)_2_, Na_2_CO_3_, DMF, 65 °C, 45% yield, (d) **21**, Pd(OAc)_2_, D^*t*^BPF, Na_2_CO_3_, NMP, 80 °C, 57% yield.

Our synthesis of (+)-pestalazine B (**4**) began with the diastereoselective azo-coupling using readily available compound **17** (Scheme 4). Under the standard reaction conditions developed with model compound **14** (Table 1), compound **17** coupled with PhN_2_BF_4_ to afford compound **18** and its diastereomer in a 6 : 1 ratio. Changing the base additive from K_2_CO_3_ to Na_2_CO_3_ while using 0.05 equiv. of **CPA8a** catalyst, compound **18** was obtained with improved diastereoselectivity (>20 : 1) and 79% yield. With **18** in hand, reductive cleavage of the N=N double bond by hydrazine hydrate and Pd/C at 80 °C proceeded smoothly to provide amino compound **19** in 80% yield. The stereochemistry of **19** was consistent with the natural product (confirmed by NOE, see ESI†). Next, we attempted to construct a C–N bond by coupling between **19** and 1,2-dihalobenzene (Table 2) but with minimal success. We initially tried Pd-catalyzed Buchwald–Hartwig amination using various ligands such as xantphos, BINAP, DPPF, Xphos, Brettphos, DPEphos and indole-derived phosphine ligands. However, only trace amounts of amination product **20** were detected by LCMS (entry 1). We then attempted copper-induced Ullmann coupling utilizing CuI or Cu(OAc)_2_ and ligands such as 2-isobutyrylcyclohexanone or 2,2,6,6-tetramethylheptane-3,5-dione (TMHD). Unfortunately, these yields were still unacceptable (entry 2).27*a* Success in such reactions would rely on two factors: selective amination on different amine derivatives and the basicity of the reaction solutions. Traditionally, amination with amides as substrates will proceed more easily than with hindered amines. In addition, the strong base used in the reaction mixtures will result in the racemization of compound **19**. Since hypervalent iodine reagents were widely used for C–N formation under neutral or weakly basic conditions, we decided to pursue this strategy and explore *ortho*-brominated hypervalent iodine reagents for formation of the C–N bond of our target (entries 5–11). Under the optimized conditions of using hypervalent iodine reagent **A3** (5.0 equiv.), Cu(OAc)_2_ (1.0 equiv.), and Na_2_CO_3_ additive (2.0 equiv.), compound **20** was obtained in 45% yield (entry 10) after heating at 80 °C in DMF solvent for 12 hours.27*b* Finally, Larock annulation of **20** and **21** with sterically bulky ligand D^*t*^BPF provided the target molecule (+)-pestalazine B (**4**) in 16.2% overall yield over this 4-step total synthesis (from known compound **17**).^25^ The NMR and CD spectral properties of (+)-pestalazine B were in excellent agreement with the natural product.8*a*

**Table 2:** Selective amination of hindered amine **18**

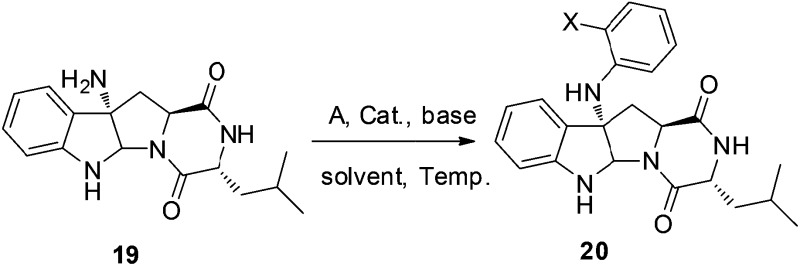						
Entry	A	Metal	Base	Solvent	*T* (°C)	Yield
1^*a*^	**A1**	Pd_2_dba_3_	*t*-BuONa	Toluene	80	Trace
2^*b*^	**A2**	CuI or Cu(OAc)_2_	K_2_CO_3_ or Li_2_CO_3_ or Cs_2_CO_3_	DMF or THF or MeCN	60	Trace
3	**A3**	CuI	Na_2_CO_3_	NMP	80	Trace
4	**A3**	CuI	Na_2_CO_3_	DMF	80	22%
5	**A3**	Cu(OAc)_2_	Na_2_CO_3_	DMF	80	34%
6	**A3**	Cu(OAc)_2_	K_2_CO_3_	DMF	80	Trace
7	**A3**	Cu(OAc)_2_	Na_3_PO_4_	DMF	80	28%
8	**A3**	Cu(OAc)_2_	Na_2_CO_3_	DMF	65	42%
9	**A3**	Cu(OAc)_2_	Na_2_CO_3_	DMF	50	26%
10	**A3**	Cu(OTf)_2_	Na_2_CO_3_	DMF	65	45%
11	**A4**	Cu(OAc)_2_	Na_2_CO_3_	DMF	80	33%
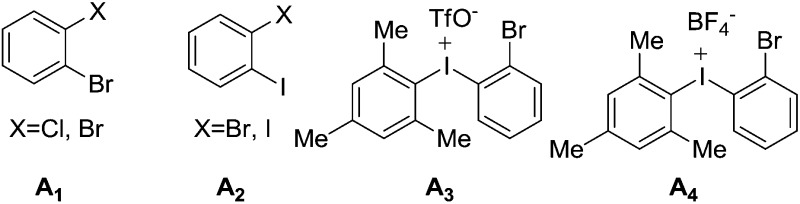						

^*a*^Ligand tested: xantphos, BINAP, DPPF, Xphos, Brettphos, DPEphos or indole-derived phosphine ligands.

^*b*^Ligand used: 2-isobutyrylcyclohexanone or TMHD.

## Conclusions

We have completed the first enantioselective total synthesis of (–)-psychotriasine (**2**) and the enantioselective total synthesis of (+)-pestalazine B (**4**) based on a unified strategy with the newly developed method. In particular, to construct the 3α-amino-hexahydropyrrolo[2,3-*b*]indole core structure, a method involving a ligand-controlled enantioselective azo-coupling of tryptamine derivatives by ion-pairing induction or *via* mixed solvent-induced diastereoselective azo-coupling of tryptophan derivatives was applied. This cascade reaction sequence provides a rapid synthetic approach to the related indole alkaloids. In addition, during our synthesis of (+)-pestalazine B (**4**), we utilized a strategy that features a sterically hindered amination process using a hypervalent iodine reagent and copper(i) catalyst. Further total syntheses of other related natural products employing this unified strategy and investigations of their biological activities are underway.

## Supplementary Material

Supplementary information

Crystal structure data
