# Supplementary material for: Enantioselective and diastereoselective azo-coupling/iminium-cyclizations: a unified strategy for the total syntheses of (–)-psychotriasine and (+)-pestalazine B
Source: Chem Sci. 2015 May 5;6(6):3599–605. doi: 10.1039/c5sc00338e (PMC5659145; doi:10.1039/c5sc00338e)
Supplement: Supplementary file 1 [file SC-006-C5SC00338E-s001.pdf]

Electronic Supplementary Material (ESI) for Chemical Science.
This journal is © The Royal Society of Chemistry 2015

Enantioselective and Diastereoselective Azo-coupling/Iminium-Cyclizations: A Unified Strategy for the Total Syntheses of (-)-Psychotriasine and (+)-Pestalazine B

Qi Li,[a,b] Tingting Xia,[b] Licheng Yao,[b] Haiteng Deng, *[,c] Xuebin Liao*[,b]

[a] *Tsinghua-Peking Joint Center for Life Sciences, Beijing 100084, China*

[b] *Department of Pharmacology and Pharmaceutical Sciences, School of Medicine, Collaborative Innovation Center for Diagnosis and Treatment of Infectious Diseases, Tsinghua University, Beijing, 100084, China*

[c] *MOE Key Laboratory of Bioinformatics, School of Life Sciences, Tsinghua University, Beijing 100084 (China)*

Supporting Information

Table of Contents

Experimental procedures and characterization data 2

X-ray structure of **16** 16

CD spectra of (+)-Pestalazine B 22

NMR spectra 23

General Methods. All commercially available reagents were used without further purification. All dry solvents such as toluene, tetrahydrofuran, dichloromethane, acetonitrile and ethyl ether were purified by solvent purification systems. Flash column chromatography was performed on silica gel (200-300 mesh). ^{1}H NMR spectra were recorded on a 400 MHz or 600 MHz NMR spectrometer and ^{13}C NMR spectra were recorded on a 100 MHz or 150 MHz NMR spectrometer. Enantiomeric ratio was measured on a Shimadzu Chiral HPLC using Chiralpak OD-H, AS-H or AD-H columns.

S-Table1. Enantioselective azo-coupling of tryptaminederivatives.[a] [b]

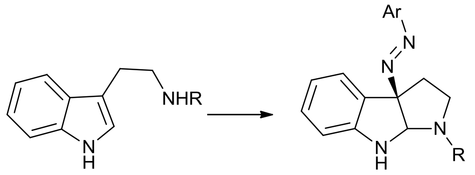

6a, R =CO_2Me
6b, R =Cbz
6c, R =Boc
6d, R =Bz

7a, R = CO_2Me, Ar = Ph
7b, R = Cbz, Ar = Ph
7c, R = Boc, Ar = Ph
7d, R = Bz, Ar = Ph
7f, R = Bz, Ar = 4-CF_3-Ph

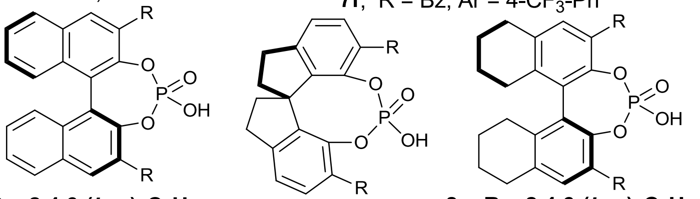

8a: R = 2,4,6-(*i*-pr)$_3C_6H_2$
8b: R = 3,5-$(CF_3)_2$-C_6H_3
8c: R = 3,5-$(CH_3)_2$-C_6H_3
8d: R = Ph_3Si
8f: R = anthryl
8e: R = 2,4,6-(*i*-pr)$_3C_6H_2$

Entry	Substrate	Solvent	CPA	Additive[c]	T (°C)	Priduct	Yield[d]	Er
1	**6a**	toluene	8a	--	0	**7a**	92%	52.5:47.5
2	**6a**	mysitylene	8a	--	0	**7a**	63%	54:46
3	**6a**	PhF	8a	--	0	**7a**	89%	50:50
4	**6a**	Et_2O	8a	--	0	**7a**	70%	64:36
5	**6a**	*t*-BuOMe	8a	--	0	**7a**	89%	67:33
6	**6b**	*t*-BuOMe	8a	--	0	**7b**	82%	61:39
7	**6c**	*t*-BuOMe	8a	--	0	**7c**	74%	64:36
8	**6d**	t-BuOMe	8a	--	0	**7d**	74%	27:73
9	**6d**	*t*-BuOMe	8a	--	-40	**7d**	60%	25:75
10	**6d**	*t*-BuOMe	8b	--	-40	**7d**	68%	62:38
11	**6d**	t-BuOMe	8c	--	-40	**7d**	54%	60:40
12	**6d**	*t*-BuOMe	8d	--	-40	**7d**	44%	58:42
13	**6d**	*t*-BuOMe	8e	--	-40	**7d**	94%	26:74
14	**6d**	*t*-BuOMe	8f	--	-40	**7d**	76%	26:74
15	**6d**	*t*-BuOMe	8a	1	-40	**7d**	60%	20:80
16	**6d**	t-BuOMe	8a	2	-40	**7d**	60%	20:80
17	**6d**	*t*-BuOMe	8a	3	-40	**7d**	91%	14.5:85.5
18	**6d**	*t*-BuOMe	8a	3	-40	**7d**	88%	9.5:90.5
19	**6d**	*t*-BuOMe	8a	3	-60	**7f**	99% (69%)[e]	7.5:92.5 (<2:98) [e]

[a] Condition A (no additive): **6** (0.1 mmol), ArN_2BF_4 (0.11 mmol), CPA catalyst (0.005 mmol) and Na_2CO_3 (0.11 mmol) in solvent (1.0 mL); Condition B (with additive): **6** (0.1 mmol), ArN_2BF_4 (0.11 mmol), CPA (0.005 mmol), additive (0.1 mL of 0.01 M stock solution in *t*-BuOMe) and Na_2CO_3 (0.11 mmol) in *t*-BuOMe (0.9 mL). [b] Entries 1-17: Ar = Ph; entries 18-19: Ar = 4-$CF_3C_6H_4$. [c] Additive 1 = pyridine, additive 2 = 2,6-lutidine, additive 3 = 2,6-di-*t*-Bu-pyridine; [d] Isolated yield; [e] Recrystallized in petroleum ether.

Synthesis of Compound 7f

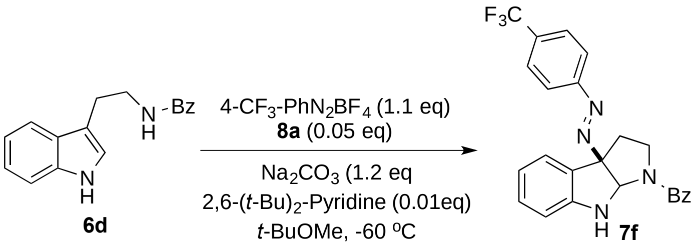

To a stirred mixture of tryptamine derivative **6d** (26.4 mg, 0.1 mmol), Na_2CO_3 (11.6 mg, 0.11mmol), **CPA1** (3.8 mg, 0.005 mmol), a solution of 2,6-di-*t*-Bupyridine (0.1 mL, 0.001 mmol, 0.01 M) in *t*-BuOMe and *t*-BuOMe (0.9 mL) was added 4-trifluoromethylbenzene diazoium tetrafluoroborate (28.6 mg, 0.11 mmol) in one portion at -60 °C. Then the suspension was kept stirring at -60 °C until **6d** was consumed (detected by TLC). The reaction mixture was then diluted with H_2O and EtOAc. The organic layer was washed with H_2O and brine, dried over Na_2SO_4, filtered and concentrated under vacuum. The resulting residue was purified by flash column chromatography (silica gel, petroleum ether/ethyl acetate = 5/1) to give azo compound **7f** as yellow oil (43.4 mg, 99% yield, er = 7.5:92.5; 30 mg, 69% yield, er < 2:98 after recrystallizing by petroleum ether).

^{1}H NMR (400 MHz, $CDCl_3$) δ 7.81 (d, *J* = 8.0 Hz, 2H), 7.72 (d, *J* = 8.0 Hz, 2H), 7.55-7.47 (m, 2H), 7.49-7.35 (m, 3H), 7.29 (d, *J* = 7.6 Hz, 1H), 7.18 (t, *J* = 7.6 Hz, 1H), 6.80 (t, *J* =7.6 Hz, 1H), 6.71 (d, *J* = 8.0 Hz, 1H), 6.38 (s, 1H), 3.82-3.72 (m, 1H), 3.63-3.50 (m, 1H), 2.65-2.54 (m, 2H); ^{13}C NMR (100 MHz, $CDCl_3$) δ 169.8, 153.7, 150.8, 135.9, 132.1 (q, *J* = 32.3 Hz), 130.32, 130.25, 128.3, 127.2, 126.3 (q, *J* = 3.7 Hz), 125.8,

125.1, 122.8, 119.0, 109.7, 89.1, 77.9, 48.6, 34.8. HRMS calcd for $C_{24}H_{20}F_3N_4O$ [M+H]: 437.1589; found: 437.1587. HPLC (ChiralPak AD-H column) 70:30 (Hex/*i*-PrOH) 1 mL/min; t_{major} (14.18 min), t_{minor} (19.39 min); 7.5:92.5 er (er <2:98, recrystallized by petroleum ether).

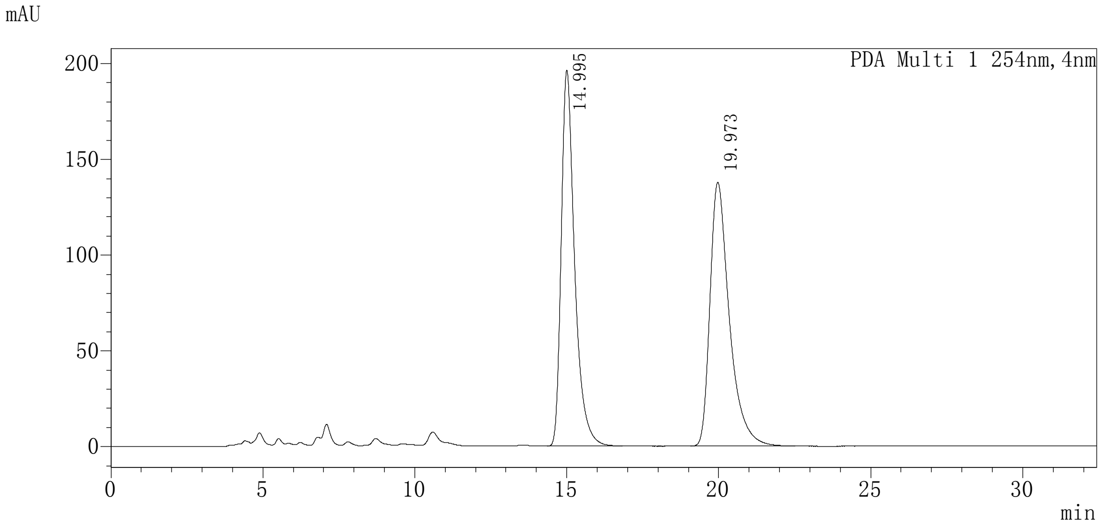

Peak #1	Retention Time (min)	Area	Height	Area Percent
1	14.995	6004225	196450	49.644
2	19.973	6090256	137687	50.356

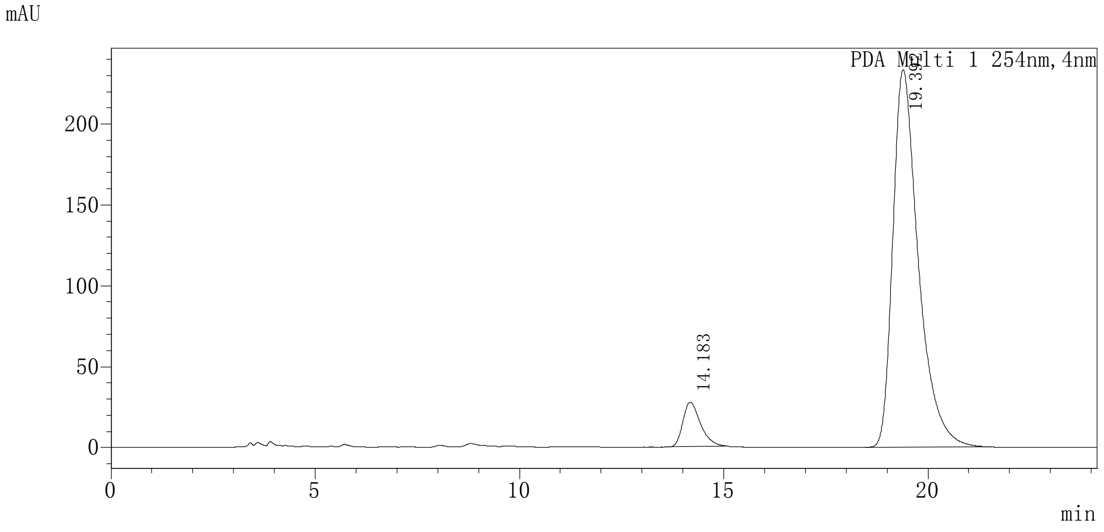

Peak #1	Retention Time (min)	Area	Height	Area Percent
1	14.183	825388	27559	7.518
2	19.392	10154095	233468	92.482

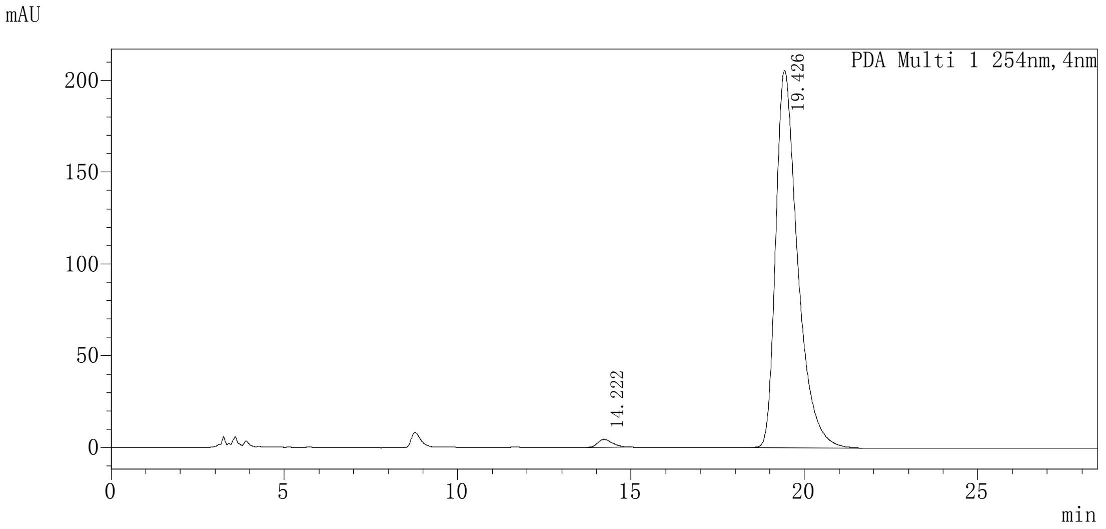

Peak #1	Retention Time (min)	Area	Height	Area Percent
1	14.222	127312	4319	1.391
2	19.426	9026995	205379	98.609

Synthesis of compound 10

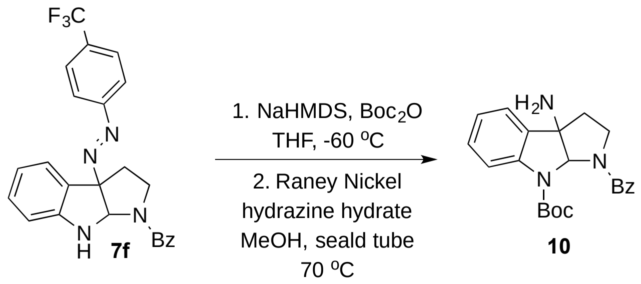

To a solution of compound **7f** (360 mg, 0.82 mmol) in THF (8 mL) was added NaHMDS (2.05 mL, 2.05 mmol, 1 M in THF) dropwise under Ar at -60 ^{o}C over 10 min. The resulting deep yellow solution was stirred for 30 min and a solution of Boc_2O (268 mg, 1.23 mmol) in THF (4 mL) was added slowly at -60 ^{o}C. Then the reaction mixture was stirred at -60 ^{o}C for one hour, quenched with saturated NH_4Cl aqueous solution,

and extracted with EtOAc. The organic layer was then washed with water and brine, dried over Na_2SO_4, concentrated and directly used without further purification.

To a solution of the above residue in MeOH (41 mL) in the sealed tube was added hydrazine hydrate (4.1 mL) and Raney Nickel (30 drops of slurry in water). After stirring at 70 ^oC for 24 h. The reaction was cooled down to rt, filtered and concentrated under vacuum and further purified by flash column chromatography (silica gel, DCM/MeOH = 50/1 - 20/1) to give amino compound **10** as white foam (276 mg, 88% yield over 2 steps)

^1H NMR (400 MHz, $CDCl_3$) δ 7.86 (d, J = 8.0 Hz, 1H), 7.60-7.53 (m, 2H), 7.49-7.33 (m, 4H), 7.33-7.27 (m, 1H), 7.07 (t, J = 7.6 Hz, 1H), 6.52 (s, 1H), 3.59 (dd, J = 11.6 Hz, 7.6 Hz, 1H), 3.19 (td, J = 12.4 Hz, 5.2 Hz, 1H), 2.19 (dd, J = 12.4 Hz, 4.8 Hz,1H), 2.16-1.95(m, 3H); ^{13}C NMR (100 MHz, $CDCl_3$) δ 171.0, 152.6, 143.0, 135.7, 131.1, 129.8, 128.6, 128.5, 123.8, 123.6, 115.4, 82.2, 82.1, 68.9, 49.2, 42.5, 28.6. HRMS calcd for $C_{22}H_{26}N_3O_3$ [M+H]: 380.1974; found: 380.1976.

Reference：

Matsuda, Y.; Kitajima, M.; Takayama, H. *Org. Lett.* **2008**, *10*, 125.

Synthesis of compound 11

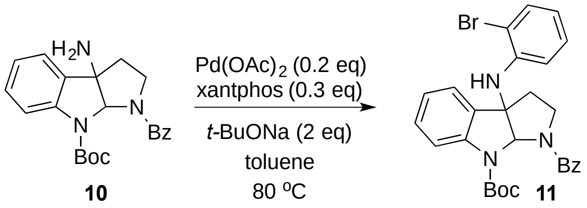

In a glovebox, a sealed tube was charged with **10** (140 mg, 0.37 mmol), 1,2-dibromobenzene (218 mg, 0.92 mmol), $Pd(OAc)_2$ (16.6 mg, 0.074 mmol), xantphos (64 mg, 0.11 mmol), *t*-BuONa (71 mg, 0.74 mmol) and toluene (7.4 mL) After stirring at 80 ^oC for 24 hrs, the reaction was diluted with EtOAc, filtered and washed with EtOAc. The resulting organic layer was washed with water and brine, dried over Na_2SO_4, concentrated under vacuum and purified by flash column chromatography (silica gel, petroleum ether/ethyl acetate = 7/1) to give compound **11** as yellow foam (139 mg, 71% yield).

^{1}H NMR (400 MHz, CD_2Cl_2) δ 7.92 (d, *J* = 8.0 Hz, 1H), 7.52-7.39 (m, 4H), 736-7.29 (m, 1H), 7.24 (d, *J* = 7.6 Hz, 1H), 7.04 (t, *J* = 7.6 Hz, 1H), 7.00-6.94 (m, 1H), 6.88 (s, 1H), 6.62-6.54 (m, 1H), 6.19 (dd, *J* = 8.0 Hz, 1.0 Hz, 1H), 4.93 (s, 1H), 3.72 (dd, *J* = 11.6 Hz, 7.6 Hz, 1H), 3.26 (td, *J* = 12.0 Hz, 5.6 Hz, 1H), 1.47(s, 9H); ^{13}C NMR (100 MHz, CD_2Cl_2) δ 171.2, 152.2, 143.7, 142.1, 135.5, 132.8, 131.1, 130.9, 129.9, 128.5, 128.4, 123.54, 123.51, 119.2, 115.0, 113.6, 111.1, 82.1, 77.0, 71.0, 47.3, 43.6, 28.2. HRMS calcd for $C_{28}H_{28}BrN_3NaO_3$ [M+Na]: 556.1212; found: 556.1206.

Synthesis of compound 9

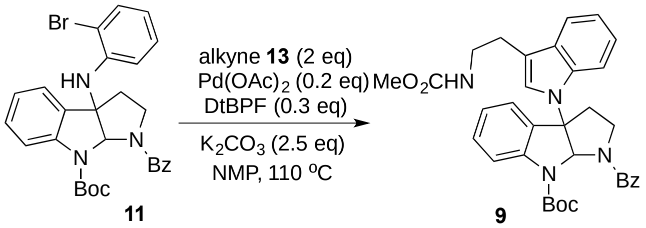

In a glovebox, a sealed tube was charged with compound **11** (137 mg, 0.26 mmol), alkyne **13** (102 mg, 0.52 mmol), $Pd(OAc)_2$ (11.5 mg, 0.052 mmol), D^{t}BPF (36.5 mg, 0.078 mmol), K_2CO_3 (89 mg, 0.66 mmol) and NMP (5.2 mL). After stirring at 110 °C for 24 hours, the reaction mixture was cooled down to rt, diluted with EtOAc, filtered and washed with EtOAc. The combined organic layer was washed with water and brine, dried over Na_2SO_4, concentrated under vacuum and purified by flash column chromatography (silica gel, petroleum ether/ethyl acetate = 2/1) to give compound **9** as yellow foam (124 mg, 83% yield).

^{1}H NMR (400 MHz, CD_2Cl_2) δ 7.97 (d, *J* = 8.0 Hz, 1H), 7.68-7.55 (m, 3H), 7.52-7.47 (m, 1H), 7.47-7.40 (m, 2H), 7.40-7.33 (m, 1H), 7.23 (s, 1H), 7.16-7.00 (m, 6H), 7.00-6.94 (m, 1H), 4.89 (s, 1H), 3.84 (dd, *J* = 11.6 Hz, 7.2 Hz, 1H), 3.62 (s, 3H), 3.50-3.38 (m, 2H), 3.33 (td, *J* = 12.0 Hz, 4.4 Hz, 1H), 3.02 (td, *J* = 12.0 Hz, 7.2 Hz, 1H), 2.91 (t, *J* = 7.2 Hz, 2H), 2.52 (dd, *J* = 12.0 Hz, 4.4 Hz, 1H), 1.48 (s, 9H); ^{13}C NMR (100 MHz, CD_2Cl_2) δ 171.2, 157.0, 152.0, 143.5, 135.8, 135.2, 131.2, 130.5, 130.0, 129.6, 128.51, 128.50, 124.2, 124.0, 123.5, 122.3, 119.8, 119.3, 115.5, 112.7, 111.3, 82.4, 78.4, 73.7, 51.9, 48.5,41.4, 40.1, 28.1, 25.8. HRMS calcd for $C_{34}H_{36}N_4NaO_5$ [M+Na]: 603.2583; found: 603.2604.

Reference:

Shen, M.; Li, G.; Lu, B. Z.; Hossain, A.; Roschangar, F.; Farina, V. C.; Senanayake, H. *Org. Lett.* **2004**, *6*, 4129.

Synthesis of compound 12

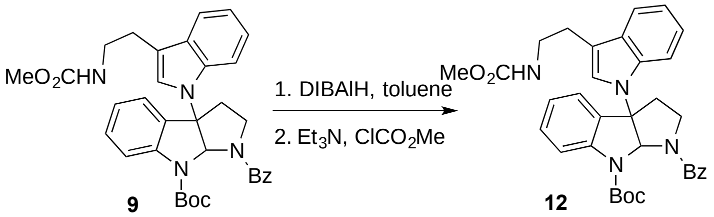

To a solution of compound **9** (102 mg, 0.18 mmol) in toluene (3.6 mL) was added DIBAl-H (0.35 mL, 0.53 mmol) dropwise at -78 ^{o}C under Argon. The reaction mixture was stirred at -78 ^{o}C until **8** was consumed completely. Then the reaction was quenched with Rochelle salt, stirred for 2 h and diluted with EtOAc. The organic layer was washed with water and brine, dried over Na_2SO_4, concentrated under vacuum and directly used for next step without further purification.

To a mixture of the above residue and Na_2CO_3 (56 mg, 0.53 mmol) in DCM-H_2O (3.6 mL-1.8 mL) was added $ClCO_2Me$ (50 mg, 0.53 mmol) dropwise at rt. After stirring at rt for 2 h, the mixture was diluted with DCM and H_2O. Then the organic layer was washed with water and brine, dried over Na_2SO_4, concentrated and purified by flash column chromatography (silica gel, petroleum ether/ethyl acetate = 2/1) to give compound **12** as yellow foam (60 mg, 64% yield over 2 steps).

^{1}H NMR (400 MHz, CD_2Cl_2) δ 7.82 (d, *J* = 8.4 Hz, 1H), 7.61-7.53 (m, 1H), 7.41-7.32 (m, 1H), 7.21 (d, *J* = 7.6 Hz, 1H), 7.14-7.00 (m, 5H), 6.69 (s, 1H), 4.87 (s, 1H), 4.14 (dd, *J* = 11.2 Hz, 7.2 Hz, 1H), 3.71 (s, 1H), 3.60 (s, 1H), 3.41 (d, *J* = 13.2 Hz, 6.8 Hz, 2H), 3.11 (td, *J* = 12.0 Hz, 7.6 Hz, 1H), 3.00 (td, *J* = 12.0 Hz, 4.8 Hz, 1H), 2.88 (t, *J* = 12.0 Hz, 2H), 2.57 (dd, *J* = 11.6Hz, 4.4Hz, 1H), 1.52 (s, 9H); ^{13}C NMR (100 MHz, CD_2Cl_2) δ 157.0, 155.1, 152.1, 143.3, 135.6, 130.5, 130.0, 129.9, 124.4, 124.2, 123.7, 122.2, 119.8, 119.4, 116.6, 112.5, 111.3, 82.2, 80.1, 74.2, 52.8, 51.9, 45.4, 41.4, 37.6, 28.1, 25.8. HRMS calcd for $C_{29}H_{34}N_4NaO_6$ [M+Na]: 557.2376; found: 557.2371.

Reference:

Gutzwiller, J.; Uskokovic, M. *J. Am. Chem. Soc.* **1970**, *92*, 204.

Synthesis of (-)-psychotrimine

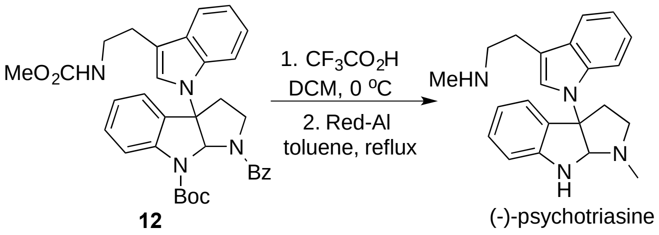

To a solution of compound **12** (70 mg, 0.13 mmol) in DCM (2.6 mL) was added CF_3CO_2H (1.3 mL) at 0 °C, and the mixture was stirred for one hour. Then the reaction was diluted with DCM, washed with H_2O and brine, dried over Na_2SO_4, filtered, concentrated under vacuum and directly used for the next step without further purification.

To a solution of the above residue in toluene (2.6 mL) was added Red-Al (0.45 mL, 1.57 mmol, 3.5 M in toluene) dropwise at rt over 10 min and the mixture was heated to reflux for 1 hour. Then the reaction was quenched with Rochelle salt, stirred for 2 hours and diluted with DCM. The organic layer was washed with H_2O and brine, dried over Na_2SO_4, filtered, concentrated under vacuum and purified by flash column chromatography (silica gel, DCM/MeOH/ammonium hydroxide = 100/5/1 to DCM/MeOH = /5/1) to give (-)-psychotrimine (**2**) as white foam (23 mg, 51% yield over 2 steps).

^{1}H NMR (400 MHz, CD_3OD) δ 7.52 (dd, *J* = 6.8 Hz, 1.2 Hz, 1H), 7.38 (s, 1H), 7.13 (d, *J* = 7.6 Hz, 1H), 7.05 (td, *J* = 7.6 Hz, 1.2 Hz, 1H), 7.00-6.90 (m, 2H), 6.85 (d, *J* = 7.6 Hz, 1H), 6.68 (d, *J* = 8.0 Hz, 1H), 6.56 (t, *J* = 7.6 Hz, 1H), 5.21 (s, 1H), 3.26-3.14 (m, 1H), 3.00-2.89 (m, 3H), 2.89-2.81 (m, 2H), 2.65-2.54 (m, 1H), 2.51-2.41 (m, 1H), 2.45 (s, 3H), 2.38 (s, 3H); ^{13}C NMR (100 MHz, CD_3OD) δ 152.5, 137.7, 131.5, 130.9, 130.6, 125.1, 124.7, 122.4, 120.1, 119.7, 119.5, 113.05, 113.02, 110.1, 87.1, 77.5, 52.9, 52.2, 40.0, 36.4, 35.9, 25.8. HRMS calcd for $C_{22}H_{27}N_4$ [M+H]: 347.2236; found: 347.2232; $[\alpha]_D^{30}$ = –138 °(c 0.3, MeOH).

Reference:

Newhouse, T.; Baran, P. S. *J. Am. Chem. Soc.* **2008**, *130*, 10886.

Synthesis of compound 16

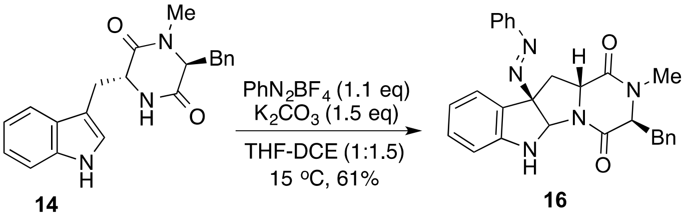

To a suspension of **14** (34.7 mg, 0.1 mmol) and K_2CO_3 (20.7 mg, 0.15 mmol) in THF-DCE (1:1.5, 1 mL) was added PhN_2BF_4 (21.1 mg, 0.11 mmol) in one portion at 15 ^{o}C. After stirring for 48 h at 0 ^{o}C, the reaction was diluted with EtOAc and H_2O. Then the organic layer was washed with H_2O and brine, dried over Na_2SO_4, filtered and then concentrated under vacuum and further purified by flash column chromatography (silica gel, DCM/petroleum ether/ethyl acetate = 1/1/1) to give compound **16** as yellow foam (27.5 mg, 61% yield).

^{1}H NMR (400 MHz, CD_2Cl_2) δ 7.76-7.67 (m, 2H), 7.56-7.49 (m, 3H), 7.22-7.08 (m, 6H), 7.02 (t, *J* = 7.2 Hz, 1H), 6.74 (t, *J* = 8.4 Hz, 1H), 6.65 (d, *J* = 8.0 Hz, 1H), 6.07 (s, 1H), 4.19 (t, *J* = 4.4 Hz, 1H), 3.25 (dd, *J* = 14.0 Hz, 4.0 Hz, 1H), 3.11 (dd, *J* = 14.0 Hz, 4.8 Hz, 1H), 3.00-2.91 (m, 4H), 2.44 (dd, *J* = 11.6 Hz, 6.4 Hz, 1H), 2.16 (dd, *J* = 13.2 Hz, 12.0 Hz, 1H); ^{13}C NMR (100 MHz, CD_2Cl_2) δ 166.6, 166.3, 151.5, 148.4, 135.3, 131.4, 129.85, 129.82, 129.1, 128.8, 127.8, 127.6, 124.4, 122.6, 119.3, 109.9, 87.5, 79.7, 65.9, 56.4, 39.4, 36.7, 32.2. HRMS calcd for $C_{27}H_{26}N_5O_2$ [M+H]: 452.2087; found: 452.2096.

Synthesis of alkyne 21

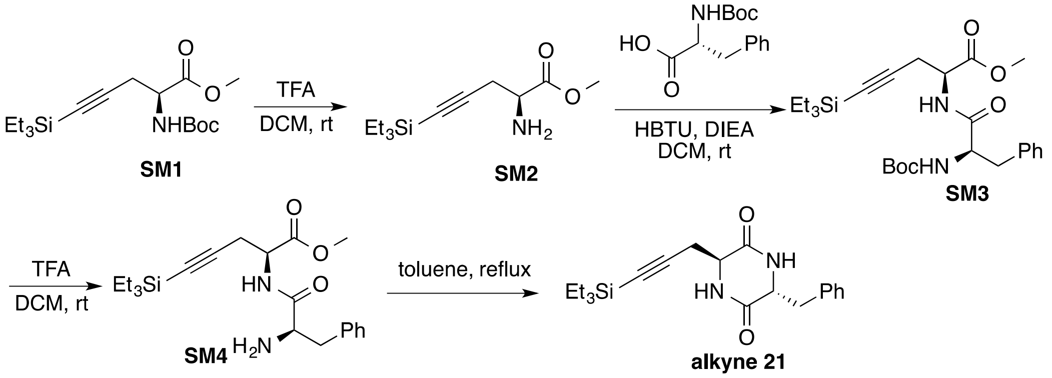

To a solution of **SM1** (3.3g, 9.7 mmol) in DCM (35 mL) was added TFA (15 mL, 195 mmol) dropwise at 0 °C, then the cooling bath was removed and the reaction mixture was left to stir overnight. The reaction was quenched by sodium hydroxide aqueous solution under 0℃, adjusted pH to 8~9, and extracted with EtOAc (3 x 50 mL). The combined organic layer was washed with H_2O and brine, dried over Na_2SO_4, filtered and concentrated. The residue **SM2** was used for the next step without purification.

To a solution of **SM2** (~ 9 mmol), (D)-Tyr-Boc(2.9 g, 10.9 mmol) and HBTU (4.1g, 10.9 mmol) in DCM (100 mL) was added DIEA (3 mL, 23 mmol) dropwise at 0 °C. After stirring at rt overnight, the reaction was quenched by water, extracted with EtOAc (3 x 50 mL). The combined organic layer was washed with H_2O and brine, dried over Na_2SO_4, filtered and concentrated. The residue was then filtered through silica gel pad using EtOAc/PE(1/4) as eluent to yield **SM3** (4.3g, 98%).

To a solution of **SM3** in DCM (30 mL) was added TFA (14 mL, 180 mmol) slowly at 0 °C, then the reaction mixture was stirred at rt overnight. The reaction was quenched by sodium hydroxide aqueous solution at 0℃, adjusted pH to 8~9, and extracted with EtOAc (3 x 50 mL). The combined organic layer was washed with H_2O and brine, dried over Na_2SO_4, filtered and concentrated. The crude **SM4** was dissolved in toluene (130 mL) and the resulting solution was refluxed for 24 hours. The precipitated white solid was filtered and washed with toluene (3 x 20 mL) to get **alkyne 21** without further purification (2.8 g, 7.7 mmol, 80% yield).

^{1}H NMR (400 MHz, DMSO-*d6*) δ 8.35 (s, 1H), 8.02 (s, 1H), 7.31-7.20 (m, 3H),

7.20-7.12 (m, 2H), 4.15 (m, 1H), 3.22-3.08 (m, 2H), 2.89 (dd, *J* = 13.6 Hz, 4.8 Hz, 1H), 2.63 (dd, *J* = 17.2 Hz, 3.2 Hz, 1H), 2.41 (dd, *J* = 17.2 Hz, 4.8Hz, 1H), 0.91 (t, *J* = 8.0 Hz, 9H), 0.50 (q, *J* = 8.0 Hz, 6H); ^{13}C NMR (100 MHz, DMSO-*d6*) δ 166.6, 166.0, 135.8, 130.1, 128.0, 126.6, 103.9, 83.9, 38.4, 24.8, 7.2, 3.9. HRMS calcd for $C_{20}H_{29}N_2O_2Si$ [M+H]: 357.1998; found: 357.2005.

Reference:

Newhouse, T.; Lewis, C. A.; Eastman, K. J.; Baran, P. S. *J. Am. Chem. Soc.* **2010**, *132*, 7119.

Synthesis of Compound 18

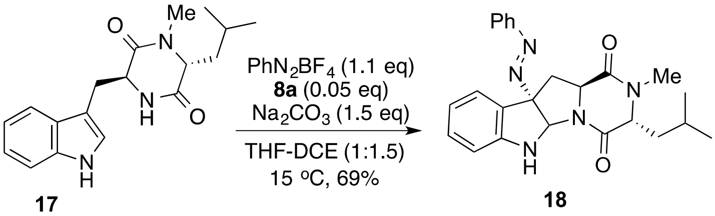

To a suspension of **17** (599 mg, 2.0 mmol), **8** (75.3 mg, 0.1 mmol) and Na_2CO_3 (233.2 mg, 2.2 mmol) in DCE-THF (1.5:1, 20mL) was added PhN_2BF_4 (461mg, 2.4mmol) at 0 °C. After stirring for 12 hours at 0℃. The reaction mixture was diluted with H_2O and extracted with EtOAc three times. The combined organic layer was washed with H_2O and brine, dried over Na_2SO_4, filtered, and concentrated under vacuum. The residue was purified by flash column chromatography (silica gel, DCM/MeOH= 80/1) to give compound **18** as yellow foam (553 mg, 69% yield).

^{1}H NMR (400 MHz, $CDCl_3$) δ 7.80-7.73 (m, 2H), 7.59 (d, *J* = 4.0 Hz, 1H), 7.51-7.43 (m, 3H), 7.27 (d, *J* = 7.2 Hz, 1H), 7.16 (td, *J* = 8.0 Hz, 1.2 Hz, 1H), 6.78 (td, *J* = 7.6 Hz, 0.4 Hz, 1H), 6.68 (d, *J* = 8.0 Hz, 1H), 6.29 (d, *J* = 4.0 Hz, 1H), 5.74 (d, *J* = 4.0 Hz, 1H), 4.23 (dd, *J* = 11.2 Hz, 6.8 Hz, 1H), 4.03-3.90 (m, 1H), 3.30 (dd, *J* = 13.6 Hz, 6.8 Hz, 1H), 2.53 (dd, *J* = 13.6 Hz, 11.2 Hz, 1H), 1.86-1.74 (m, 1H), 1.70-1.60 (m, 2H); ^{13}C NMR (100 MHz, $CDCl_3$) δ 169.2, 168.0, 151.4, 148.2, 131.7, 130.1, 129.3, 127.6, 124.3, 122.9, 119.5, 110.4, 87.5, 80.8, 57.2, 56.3, 42.5, 38.9, 24.5, 23.3, 21.4. HRMS calcd for $C_{23}H_{26}N_5O_2$ [M+H]: 404.2087; found: 404.2076.

Reference:

Perez-Balado, C.; De Lera, A. R. *Org. Biomol. Chem.* **2010**, *8*, 5179.

Synthesis of compound 19

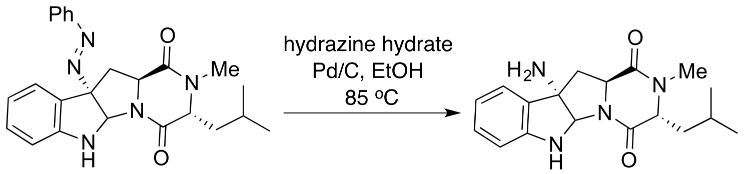

18 **19**

A mixture of **18** (101 mg, 0.25 mmol), 10 wt. % Pd/C (133 mg, 0.5 mmol) and hydrazine hydrate (125 uL, 2.5 mmol) in ethanol (4 mL) was stirred for 1 hour at 85 °C. Then the mixture was filtered through celite, washed with ethyl alcohol and concentrated. The residue was purified by flash column chromatography (silica gel, DCM/MeOH = 40/1) to give compound **19** as a white solid. (63 mg, 80% yield)

^{1}H NMR (400 MHz, DMSO-*d6*) δ 8.41 (d, *J* = 4.4 Hz, 1H), 7.20 (d, *J* = 7.2 Hz, 1H), 6.98 (t, *J* = 7.6 Hz, 1HG), 6.62 (t, *J* = 7.6 Hz, 1H), 6.56 (d, *J* = 4.0 Hz, 1H), 6.49 (d, *J* = 8.0 Hz, 1H), 5.21 (d, *J* = 4.0 Hz, 1H), 4.64 (dd, *J* = 11.2 Hz, 6.4 Hz, 1H), 3.74-3.64 (m, 1H), 2.44 (brs, 2H), 2.29 (dd, *J* = 12.8 Hz, 6.4 Hz, 1H), 1.81-1.61 (m, 3H), 1.53-1.42 (m, 1H), 0.95 (d, *J* = 6.4 Hz, 3H), 0.91 (d, *J* = 6.4 Hz, 3H); ^{13}C NMR (100 MHz, DMSO-*d6*) δ 169.1, 167.6, 147.9, 133.7, 128.2, 122.1, 117.6, 109.3, 84.9, 67.9, 57.4, 55.3, 42.4, 41.6, 23.9, 22.9, 21.4. HRMS calcd for $C_{17}H_{23}N_4O_2$ [M+H]: 315.1821; found: 315.1832.

Synthesis of compound 20

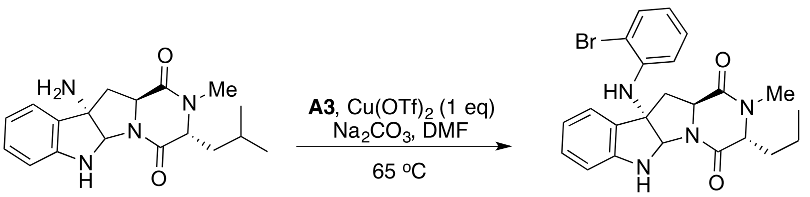

19 **19**

To an oven-dried sealed tube was charged with **19** (31.4 mg, 0.1mmol), **A3** (275.5 mg, 0.5 mmol), $Cu(OTf)_2$ (36.2 mg, 0.1 mmol), Na_2CO_3 (21.1 mg, 0.2 mmol) and DMF (1.4 mL) in a glovebox. After stirring at 65 °C under N_2 for12 hours, the reaction was quenched by H_2O and extracted by EtOAc. The combined organic layer was washed

with H_2O and brine, dried over Na_2SO_4, filtered and concentrated under vacuum. The residue was purified by flash column chromatography (silica gel, acetone/petroleum = 1/2) to give compound **20** as a pale yellow solid (21.3 mg, 45% yield).

^{1}H NMR (400 MHz, $CDCl_3$) δ 7.43 (d, *J* = 8.0 Hz, 1H), 7.15 (t, *J* = 8.0 Hz, 1H, 7.10 (d, *J* = 7.2 Hz, 1H), 6.91 (t, *J* = 7.6 Hz, 1H), 6.74 (t, *J* = 7.6 Hz, 1H), 6.66 (d, *J* = 8.0 Hz, 1H), 6.62-6.53 (m, 2H), 6.16 (d, *J* = 8.4 Hz, 1H), 5.96 (d, *J* = 4.0 Hz, 1H), 5.44 (d, *J* = 3.6 Hz, 1H), 4.86 (s, 1H), 4.63 (dd, *J* = 12.0 Hz, 6.0 Hz, 1H), 4.01-3.92 (m, 1H), 2.82 (dd, *J* = 14.0 Hz, 6.0 Hz, 1H), 2.50 (dd, *J* = 13.6 Hz, 12.0 Hz, 1H), 1.86-1.74 (m, 1H), 1.67 (t, *J* = 7.2 Hz, 2H), 1.00 (d, *J* = 6.4 Hz, 3H), 0.96 (d, *J* = 6.4 Hz, 3H); ^{13}C NMR (100 MHz, $CDCl_3$) δ 169.2, 168.0, 151.4, 148.2, 131.7, 130.1, 129.3, 127.6, 124.3, 122.9, 119.5, 110.4, 87.5, 80.8, 57.2, 56.3, 42.5, 38.9, 24.5, 23.3, 21.4. HRMS calcd for $C_{23}H_{25}N_4O_2NaBr$ [M+Na]: 491.1059; found: 491.1061.

Reference:

(a) Ley, S. V.; Thomas, A. W.; *Angew. Chem. Int. Ed.* **2003**, *42*, 5400;

(b) Shafir, A. P.; Lichtor, A.; Buchwald, S. L. *J. Am. Chem. Soc.* **2007**, *129*, 3490.

Synthesis of (+)-pestalazine B (4)

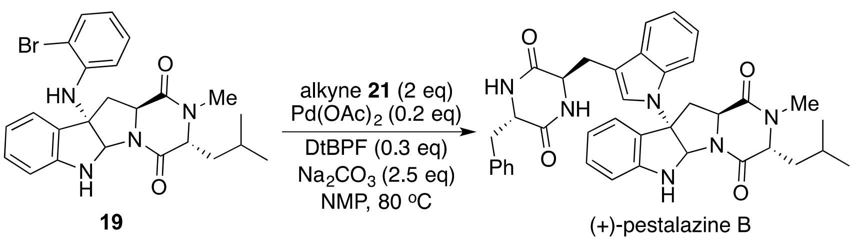

In a glovebox, a sealed tube was charged with compound **19** (70.4 mg, 0.15 mmol), alkyne **21** (107 mg, 0.30 mmol), $Pd(OAc)_2$ (6.7 mg, 0.030 mmol), D^{t}BPF (21.3 mg, 0.045 mmol), Na_2CO_3 (39.8 mg, 0.37 mmol), and NMP (2.1 mL). After stirring at 80 °C for 3 hours. The reaction mixture was cooled down to rt, diluted with EtOAc, filtered and washed with EtOAc. The resulting organic layer was stirred with 2 M HCl vigorously, washed with water and brine, dried over Na_2SO_4, concentrated and purified by flash column chromatography (silica gel, petroleum ether/acetone = 2/1 to 1.5/1) to give (+)-pestalazine B (**4**) as white solid (51.7 mg, 57% yield).

^{1}H NMR (600 MHz, acetone-*d*6) δ 7.69-7.61 (m, 2H), 7.53-7.48 (m, 1H), 7.25-

7.15 (m, 4H), 7.12-7.06 (m, 2H), 7.00-6.90 (m, 4H), 6.85 (dt, *J* = 5.2 Hz, 0.4 Hz, 1H), 6.79 (dt, *J* = 5.6 Hz, 0.8 Hz, 1H), 6.76 (s, 1H), 6.64 (td, *J* = 5.6 Hz, 0.8 Hz, 1H), 6.58 (d, *J* = 2.4 Hz, 1H), 6.05 (d, *J* = 4 Hz, 1H), 4.85 (dd, *J* = 4 Hz, 7.6Hz, 1H), 3.91 (quint, *J* = 3.2 Hz, 1H), 3.72-3.65 (m, 2H), 3.51 (t, *J* = 3.6 Hz, 1H), 3.23, (d, *J* = 3.6 Hz, 2H), 3.05 (dd, *J* = 9.2 Hz, 3.6 Hz, 1H), 2.99 (dd, *J* = 9.2 Hz, 3.2 Hz, 1H), 2.47 (dd, *J* = 10.0 Hz, 8.0 Hz, 1H), 1.75-1.67 (m, 1H), 1.58-1.47 (m, 1H), 0.95 (d, *J* = 4.4 Hz, 3H), 0.90 (d, *J* = 4.4 Hz): ^{13}C NMR (150 MHz, acetone-*d*6) δ 168.9, 168.52, 168.49, 148.8, 137.0, 136.6, 130.8, 130.7, 129.8, 129.1, 127.6, 126.48, 126.45, 123.5, 122.4, 120.5, 120.27,120.26, 119.7, 112.9, 111.0, 83.4, 76.4, 57.30, 57.26, 56.2, 55.8, 25.1, 23.2, 21.8. HRMS calcd for $C_{37}H_{39}N_6O_4$ [M+1]: 631.3033; found: 631.3007. $[\alpha]_D^{25}$ = +204 °(c 0.2, MeOH).

Reference:

Shen, M.; Li, G.; Lu, B. Z.; Hossain, A.; Roschangar, F.; Farina, V. C.; Senanayake, H. *Org. Lett.* **2004**, *6*, 4129.

Thermal ellipsoid plot of the X-ray structure of compound **16** at the 50% probability level.

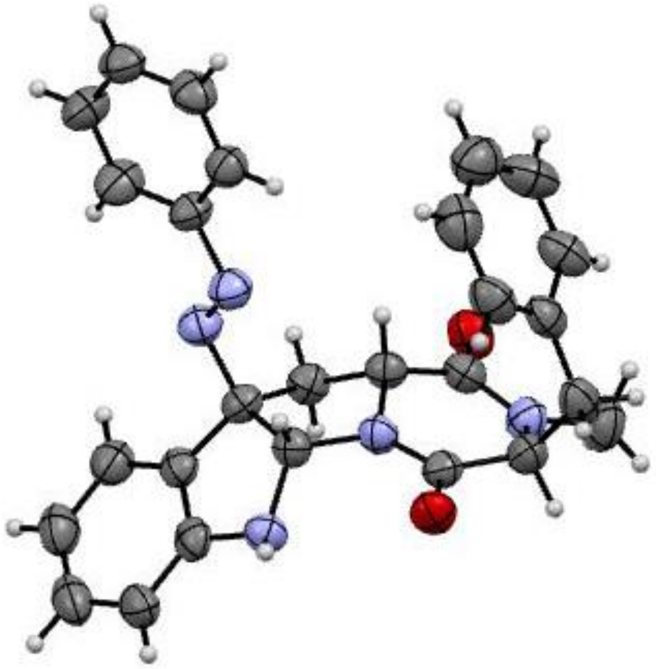

Table 1. Details of Data Collection, Processing and Structure Refinement

Sample code	**LiaoXB-1**
Molecular formula	$C_{27}H_{25}N_5O_2$
Molecular weight	451.52
Color and habit	yellow prism
Crystal size	0.3 × 0.3 × 0.6 mm
Crystal system	orthorhombic
Space group	$P2_12_12_1$ (No. 19)
Unit cell parameters	*a* = 11.295(5) Å α = 90.00°<br>*b* = 12.390(4) Å β = 90.00°<br>*c* = 16.088(3) Å γ = 90.00°<br>*V* = 2251.5(13) Å^3 *Z* = 4 *F*(000) = 952
Density (calcd)	1.332 g/cm^3
Diffractometer	Bruker P4
Radiation	graphite-monochromatized Mo K_α, λ = 0.71073 Å
Temperature	295±2K

Scan type	ω-scan
Data collection range	$-1 < h < 13$, $-1 < k < 15$, $-19 < l < 1$; $\theta_{max} = 25.5°$
Reflections measured	Total: 3065 Unique (n): 2882 Observed [$I \geq 2\sigma(I)$]: 2321
Absorption coefficient	0.087 mm^{-1}
No. of variables, p	312

Weighting scheme $w = \dfrac{1}{\sigma^2(F_o^2) + (0.001P)^2 + 1.0P}$ $P = (F_o^2 + 2F_c^2)/3$

$R1 = \dfrac{\Sigma||F_o|-|F_c||}{\Sigma|F_o|}$ (for all reflections) 0.0602 0.0412 (for observed data)

$wR2 = \sqrt{\dfrac{\Sigma[w(F_o^2 - F_c^2)^2]}{\Sigma w(F_o^2)^2}}$ (for all reflections) 0.0849 0.0739 (for observed data)

Goof = S = $\sqrt{\dfrac{\Sigma[w(F_o^2 - F_c^2)^2]}{n - p}}$ 1.038

Largest and mean Δ/σ 0.001, 0.000

Residual extrema in final difference map -0.213 to 0.158 e $Å^{-3}$

Table 2. Atomic coordinates and equivalent isotropic temperature factors* (Å^2)

Atoms	x	y	z	$U_{eq.}$
O(1)	0.00152(15)	0.46553(15)	0.88800(11)	0.0544(5)
O(2)	0.23937(17)	0.46238(16)	0.60063(10)	0.0590(5)
N(1)	0.17082(18)	0.49082(16)	0.81727(12)	0.0450(5)
N(2)	0.06593(19)	0.44633(19)	0.66860(13)	0.0531(6)
N(3)	0.18768(19)	0.63507(18)	0.91949(13)	0.0483(6)
N(4)	0.4621(2)	0.50540(17)	0.86669(13)	0.0505(6)
N(5)	0.43884(19)	0.41377(17)	0.88923(14)	0.0522(6)
C(1)	0.0568(2)	0.4602(2)	0.82334(16)	0.0452(6)
C(2)	-0.0010(2)	0.4221(2)	0.74388(17)	0.0529(7)
C(3)	0.1841(2)	0.4582(2)	0.66549(16)	0.0471(7)
C(4)	0.2468(2)	0.4670(2)	0.74689(14)	0.0429(6)
C(5)	0.3349(2)	0.5593(2)	0.75160(15)	0.0470(6)
C(6)	0.3572(2)	0.5710(2)	0.84568(15)	0.0428(6)
C(7)	0.2374(2)	0.5362(2)	0.88653(15)	0.0430(6)
C(8)	0.3739(2)	0.6830(2)	0.87710(15)	0.0439(6)
C(9)	0.2763(2)	0.7133(2)	0.92328(15)	0.0468(7)
C(10)	0.2730(3)	0.8116(2)	0.96417(17)	0.0589(8)
C(11)	0.3693(3)	0.8794(2)	0.95676(19)	0.0668(9)
C(12)	0.4651(3)	0.8517(2)	0.90863(19)	0.0652(9)
C(13)	0.4687(3)	0.7531(2)	0.86867(18)	0.0585(8)
C(14)	-0.0325(2)	0.3022(2)	0.75096(19)	0.0600(8)
C(15)	0.0728(2)	0.2309(2)	0.75988(17)	0.0512(7)
C(16)	0.1154(3)	0.2017(2)	0.83635(18)	0.0635(8)
C(17)	0.2162(3)	0.1408(3)	0.8438(2)	0.0779(10)
C(18)	0.2761(3)	0.1086(3)	0.7745(2)	0.0784(10)
C(19)	0.2352(3)	0.1369(2)	0.6984(2)	0.0756(10)
C(20)	0.1342(3)	0.1966(2)	0.69049(18)	0.0628(9)
C(21)	0.0032(3)	0.4389(3)	0.58915(18)	0.0752(10)
C(22)	0.5410(2)	0.3487(2)	0.90803(16)	0.0475(7)
C(23)	0.5187(3)	0.2436(2)	0.92742(16)	0.0513(7)
C(24)	0.6095(3)	0.1750(2)	0.94758(17)	0.0576(8)
C(25)	0.7238(3)	0.2133(2)	0.94922(17)	0.0553(8)
C(26)	0.7461(3)	0.3180(2)	0.93043(18)	0.0583(8)
C(27)	0.6555(2)	0.3864(2)	0.90993(19)	0.0617(9)

*$U_{eq.}$ defined as one third of the trace of the orthogonalized **U** tensor.

Table 3. Bond lengths (Å) and bond angles (°)

O(1)-C(1)	1.215(3)	C(8)-C(9)	1.381(4)
O(2)-C(3)	1.217(3)	C(8)-C(13)	1.386(4)
N(1)-C(1)	1.346(3)	C(9)-C(10)	1.385(4)
N(1)-C(4)	1.451(3)	C(10)-C(11)	1.379(4)
N(1)-C(7)	1.458(3)	C(11)-C(12)	1.375(4)
N(2)-C(3)	1.344(3)	C(12)-C(13)	1.381(4)
N(2)-C(2)	1.459(3)	C(14)-C(15)	1.488(4)
N(2)-C(21)	1.464(3)	C(15)-C(16)	1.370(4)
N(3)-C(9)	1.395(3)	C(15)-C(20)	1.381(4)
N(3)-C(7)	1.448(3)	C(16)-C(17)	1.372(4)
N(4)-N(5)	1.221(3)	C(17)-C(18)	1.364(5)
N(4)-C(6)	1.476(3)	C(18)-C(19)	1.353(5)
N(5)-C(22)	1.440(3)	C(19)-C(20)	1.365(4)
C(1)-C(2)	1.512(4)	C(22)-C(23)	1.363(4)
C(2)-C(14)	1.531(4)	C(22)-C(27)	1.375(4)
C(3)-C(4)	1.493(3)	C(23)-C(24)	1.371(4)
C(4)-C(5)	1.517(3)	C(24)-C(25)	1.376(4)
C(5)-C(6)	1.541(3)	C(25)-C(26)	1.356(4)
C(6)-C(8)	1.489(3)	C(26)-C(27)	1.369(4)
C(6)-C(7)	1.565(3)		
C(1)-N(1)-C(4)	124.4(2)	C(3)-C(4)-C(5)	114.2(2)
C(1)-N(1)-C(7)	123.2(2)	C(4)-C(5)-C(6)	103.1(2)
C(4)-N(1)-C(7)	111.68(19)	N(4)-C(6)-C(8)	109.5(2)
C(3)-N(2)-C(2)	124.6(2)	N(4)-C(6)-C(5)	107.7(2)
C(3)-N(2)-C(21)	117.1(2)	C(8)-C(6)-C(5)	116.2(2)
C(2)-N(2)-C(21)	117.4(2)	N(4)-C(6)-C(7)	116.53(19)
C(9)-N(3)-C(7)	109.0(2)	C(8)-C(6)-C(7)	102.9(2)
N(5)-N(4)-C(6)	114.0(2)	C(5)-C(6)-C(7)	104.2(2)
N(4)-N(5)-C(22)	114.2(2)	N(3)-C(7)-N(1)	114.0(2)
O(1)-C(1)-N(1)	122.6(2)	N(3)-C(7)-C(6)	104.9(2)
O(1)-C(1)-C(2)	121.2(2)	N(1)-C(7)-C(6)	103.38(19)
N(1)-C(1)-C(2)	116.1(2)	C(9)-C(8)-C(13)	119.9(2)
N(2)-C(2)-C(1)	114.4(2)	C(9)-C(8)-C(6)	109.6(2)
N(2)-C(2)-C(14)	112.4(2)	C(13)-C(8)-C(6)	130.4(3)
C(1)-C(2)-C(14)	109.9(2)	C(8)-C(9)-C(10)	121.1(3)
O(2)-C(3)-N(2)	123.1(3)	C(8)-C(9)-N(3)	111.1(2)
O(2)-C(3)-C(4)	120.4(2)	C(10)-C(9)-N(3)	127.8(3)
N(2)-C(3)-C(4)	116.5(2)	C(11)-C(10)-C(9)	118.3(3)
N(1)-C(4)-C(3)	114.7(2)	C(12)-C(11)-C(10)	121.2(3)
N(1)-C(4)-C(5)	101.30(19)	C(11)-C(12)-C(13)	120.4(3)

(Table 3. continued)

C(12)-C(13)-C(8)	119.1(3)	C(19)-C(20)-C(15)	120.7(3)
C(15)-C(14)-C(2)	113.4(2)	C(23)-C(22)-C(27)	119.5(3)
C(16)-C(15)-C(20)	117.9(3)	C(23)-C(22)-N(5)	115.8(2)
C(16)-C(15)-C(14)	121.6(3)	C(27)-C(22)-N(5)	124.7(2)
C(20)-C(15)-C(14)	120.4(3)	C(22)-C(23)-C(24)	120.6(3)
C(15)-C(16)-C(17)	121.0(3)	C(23)-C(24)-C(25)	119.5(3)
C(18)-C(17)-C(16)	120.0(3)	C(26)-C(25)-C(24)	120.0(3)
C(19)-C(18)-C(17)	119.7(3)	C(25)-C(26)-C(27)	120.5(3)
C(18)-C(19)-C(20)	120.6(3)	C(26)-C(27)-C(22)	119.9(3)
Hydrogen bonding			
H(3)···O(2)$^{\#1}$	2.73(3)	N(3)-H(3)···O(2)$^{\#1}$	122(2)

Symmetry transformation code: #1 (0.5-x, 1-y, 0.5+z).

Table 4. Anisotropic thermal parameters* (Å^2)

Atoms	U_{11}	U_{22}	U_{33}	U_{23}	U_{13}	U_{12}
O(1)	0.0484(10)	0.0621(11)	0.0528(10)	-0.0053(10)	0.0149(9)	-0.0042(10)
O(2)	0.0682(12)	0.0665(12)	0.0422(9)	-0.0041(10)	0.0093(10)	-0.0087(12)
N(1)	0.0431(11)	0.0491(12)	0.0428(11)	-0.0069(10)	0.0045(10)	-0.0013(11)
N(2)	0.0505(12)	0.0648(14)	0.0439(11)	-0.0031(12)	-0.0029(11)	0.0020(12)
N(3)	0.0487(12)	0.0528(12)	0.0434(11)	-0.0042(11)	0.0056(10)	0.0024(12)
N(4)	0.0514(12)	0.0490(13)	0.0510(12)	0.0047(11)	-0.0003(11)	0.0039(12)
N(5)	0.0491(12)	0.0477(12)	0.0597(13)	0.0025(12)	-0.0018(12)	0.0033(12)
C(1)	0.0419(13)	0.0435(14)	0.0504(14)	0.0002(13)	0.0018(13)	0.0042(13)
C(2)	0.0438(13)	0.0601(16)	0.0548(15)	-0.0106(14)	-0.0026(15)	0.0049(14)
C(3)	0.0532(15)	0.0412(13)	0.0470(14)	-0.0028(13)	0.0028(14)	0.0005(14)
C(4)	0.0444(13)	0.0451(13)	0.0394(12)	0.0007(12)	0.0053(13)	0.0017(13)
C(5)	0.0467(13)	0.0506(14)	0.0438(13)	0.0021(13)	0.0040(13)	-0.0015(13)
C(6)	0.0385(13)	0.0463(14)	0.0435(13)	0.0051(12)	0.0001(12)	0.0027(12)
C(7)	0.0451(13)	0.0443(13)	0.0397(12)	0.0014(12)	0.0005(12)	0.0005(13)
C(8)	0.0457(14)	0.0434(13)	0.0426(13)	0.0031(12)	-0.0067(13)	0.0015(13)
C(9)	0.0517(15)	0.0509(15)	0.0378(13)	0.0012(12)	-0.0122(13)	-0.0002(14)
C(10)	0.0627(18)	0.0607(17)	0.0534(15)	-0.0141(15)	-0.0122(15)	0.0076(17)
C(11)	0.082(2)	0.0508(16)	0.0676(18)	-0.0052(16)	-0.0257(18)	-0.0044(19)
C(12)	0.0662(18)	0.0554(17)	0.0741(19)	-0.0007(16)	-0.0139(18)	-0.0146(17)
C(13)	0.0517(16)	0.0576(16)	0.0661(18)	0.0040(15)	-0.0044(16)	-0.0010(16)
C(14)	0.0510(15)	0.0663(17)	0.0626(16)	-0.0148(16)	0.0097(17)	-0.0137(15)
C(15)	0.0554(15)	0.0432(14)	0.0550(15)	-0.0096(13)	0.0079(15)	-0.0114(13)
C(16)	0.0732(19)	0.0645(18)	0.0529(16)	-0.0117(16)	0.0112(17)	-0.0056(18)
C(17)	0.086(2)	0.076(2)	0.071(2)	-0.0010(19)	-0.013(2)	-0.010(2)
C(18)	0.0642(19)	0.068(2)	0.103(3)	-0.001(2)	0.004(2)	-0.0010(18)
C(19)	0.087(2)	0.0583(18)	0.081(2)	-0.0070(18)	0.027(2)	0.008(2)
C(20)	0.084(2)	0.0495(16)	0.0547(16)	-0.0047(14)	0.0152(18)	-0.0022(18)
C(21)	0.068(2)	0.099(2)	0.0580(17)	-0.0104(18)	-0.0181(17)	0.001(2)
C(22)	0.0435(14)	0.0459(14)	0.0531(15)	0.0050(13)	-0.0004(13)	0.0065(13)
C(23)	0.0476(15)	0.0514(15)	0.0547(16)	0.0012(14)	-0.0005(14)	-0.0005(15)
C(24)	0.0694(19)	0.0440(15)	0.0596(17)	0.0041(14)	-0.0035(17)	0.0020(15)
C(25)	0.0552(17)	0.0566(17)	0.0540(16)	-0.0009(14)	-0.0028(15)	0.0144(15)
C(26)	0.0437(15)	0.0633(17)	0.0678(18)	0.0097(16)	0.0008(15)	0.0062(16)
C(27)	0.0571(17)	0.0471(15)	0.081(2)	0.0175(16)	-0.0005(17)	-0.0021(15)

*The exponent takes the form: $-2\pi^2\Sigma\Sigma U_{ij}h_ih_j\mathbf{a}_i^*\mathbf{a}_j^*$

Table 5. Coordinates and isotropic temperature factors* (Å^2) for H atoms

Atoms	*x*	*y*	*z*	$U_{eq.}$
H(3)	0.151(2)	0.620(2)	0.9638(16)	0.058
H(2)	-0.0762	0.4612	0.7393	0.063
H(4)	0.2887	0.3992	0.7577	0.052
H(5A)	0.3018	0.6250	0.7285	0.056
H(5B)	0.4074	0.5417	0.7223	0.056
H(7)	0.2500	0.4827	0.9305	0.052
H(10)	0.2076	0.8314	0.9958	0.071
H(11)	0.3693	0.9450	0.9848	0.080
H(12)	0.5280	0.8996	0.9029	0.078
H(13)	0.5339	0.7340	0.8365	0.070
H(14A)	-0.0838	0.2920	0.7987	0.072
H(14B)	-0.0763	0.2807	0.7018	0.072
H(16)	0.0753	0.2236	0.8840	0.076
H(17)	0.2438	0.1214	0.8962	0.093
H(18)	0.3446	0.0675	0.7794	0.094
H(19)	0.2763	0.1154	0.6512	0.091
H(20)	0.1065	0.2143	0.6378	0.075
H(21A)	0.0255	0.3734	0.5613	0.113
H(21B)	0.0236	0.4997	0.5550	0.113
H(21C)	-0.0806	0.4387	0.5990	0.113
H(23)	0.4412	0.2182	0.9270	0.062
H(24)	0.5939	0.1031	0.9600	0.069
H(25)	0.7858	0.1674	0.9632	0.066
H(26)	0.8235	0.3435	0.9315	0.070
H(27)	0.6714	0.4582	0.8973	0.074

*The exponent takes the form: $-8\pi^2 U\sin^2\theta/\lambda^2$

CD spectra of Pestalazine B

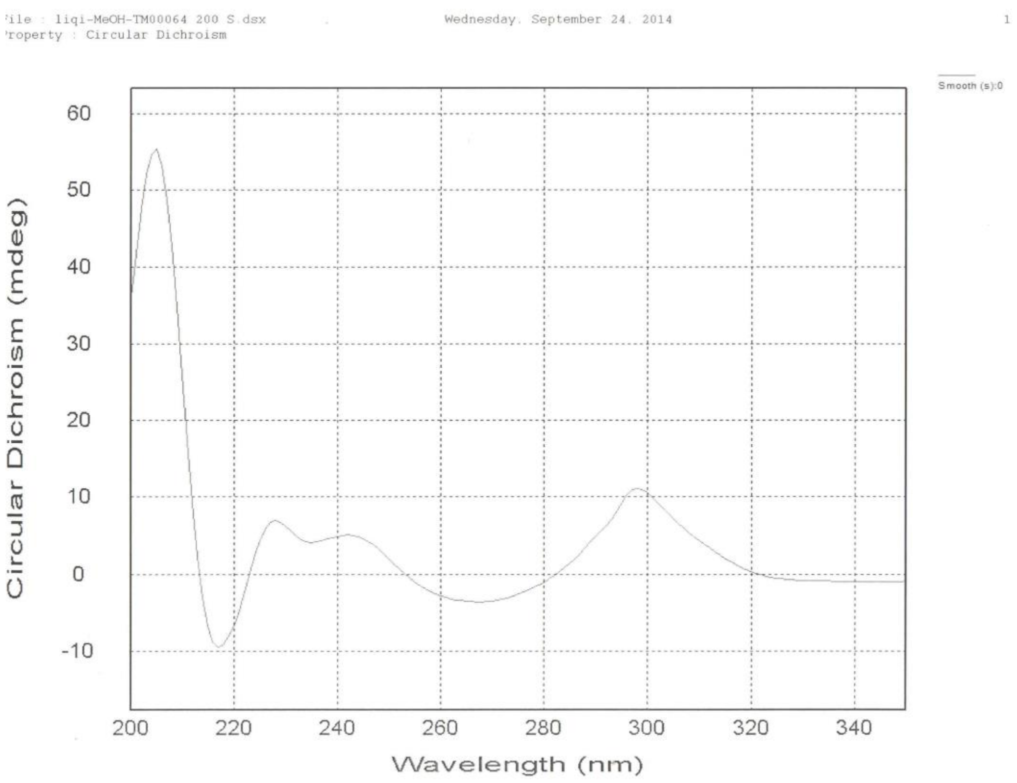

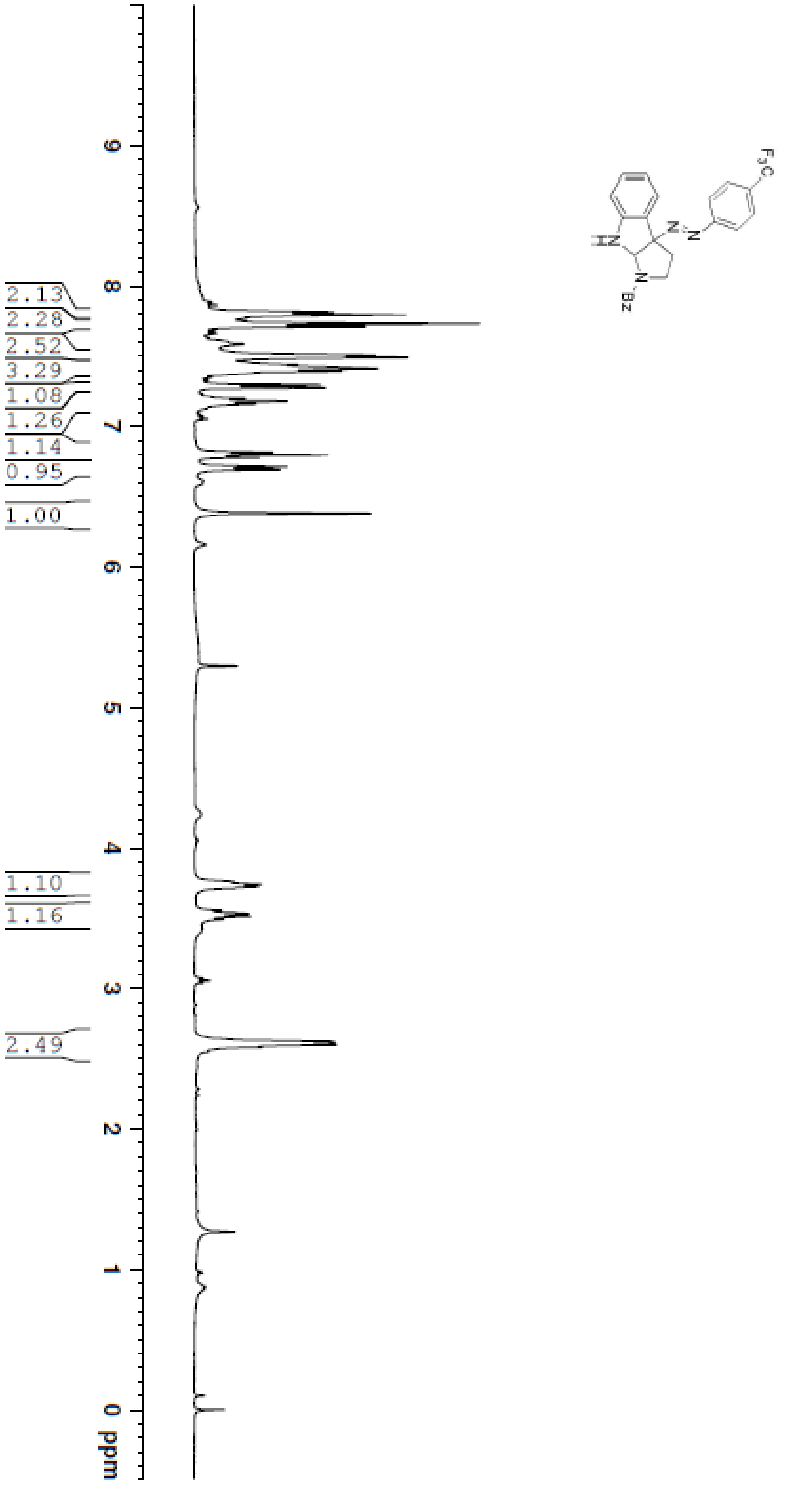
2.13
2.28
2.52
3.29
1.08
1.26
1.14
0.95
1.00
1.10
1.16
2.49
ppm
F3C
N
NH
Bz

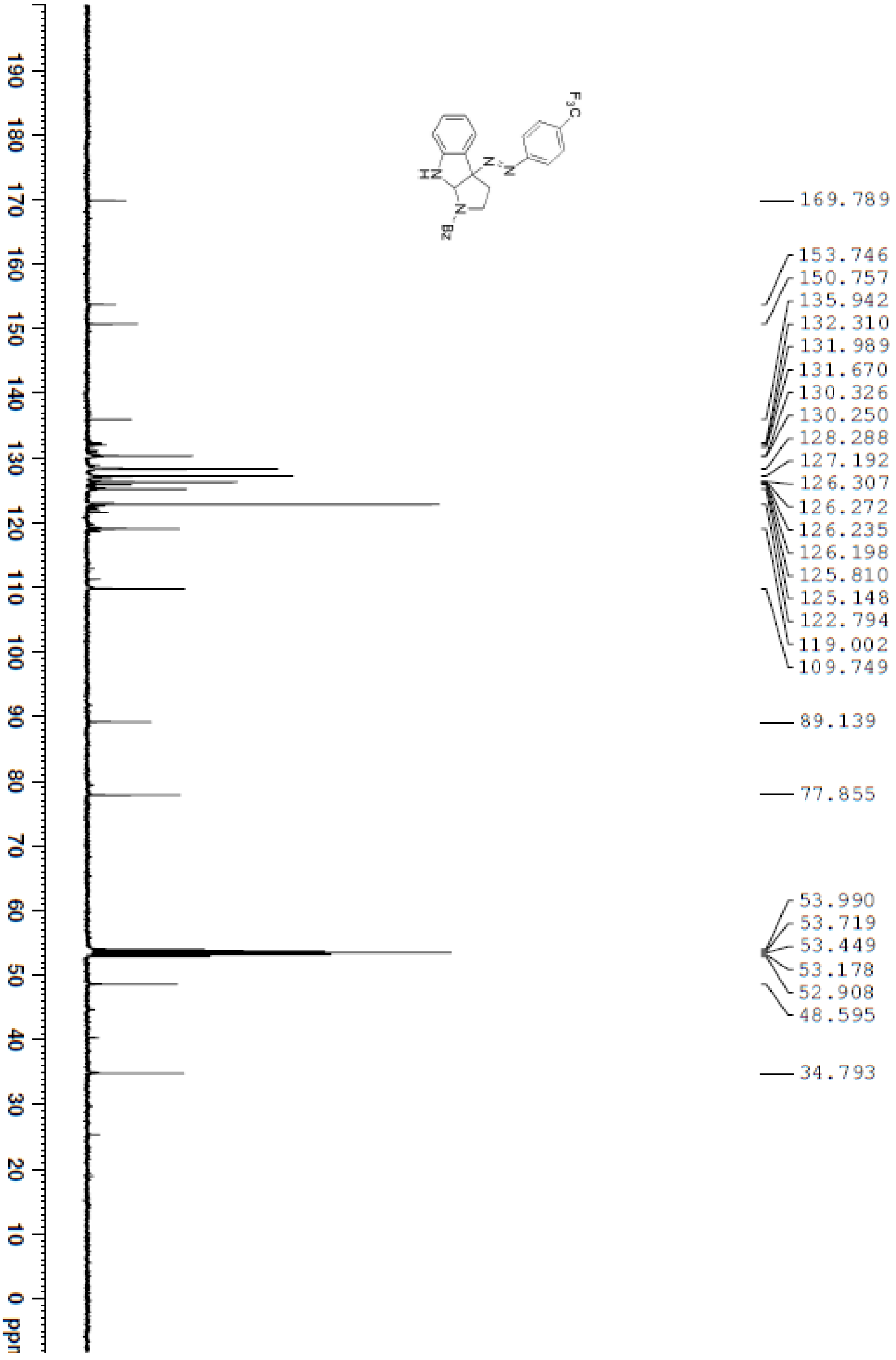
169.789
153.746
150.757
135.942
132.310
131.989
131.670
130.326
130.250
128.288
127.192
126.307
126.272
126.235
126.198
125.810
125.148
122.794
119.002
109.749
89.139
77.855
53.990
53.719
53.449
53.178
52.908
48.595
34.793
190
180
170
160
150
140
130
120
110
100
90
80
70
60
50
40
30
20
10
0 ppm
F3C
HN
N
N
N
Bz

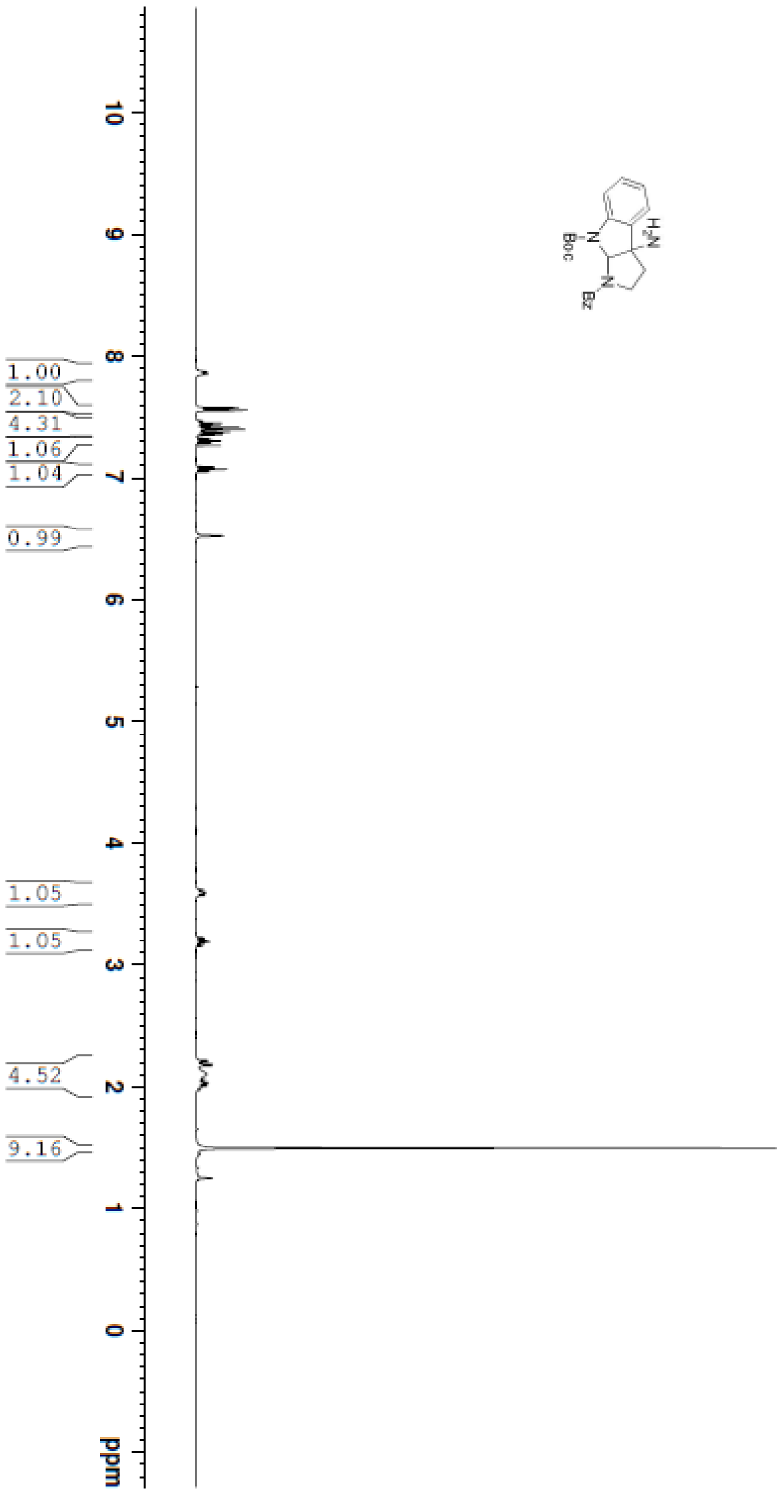
H2N
N
Boc
N
Bz
1.00
2.10
4.31
1.06
1.04
0.99
1.05
1.05
4.52
9.16
10
9
8
7
6
5
4
3
2
1
0
ppm

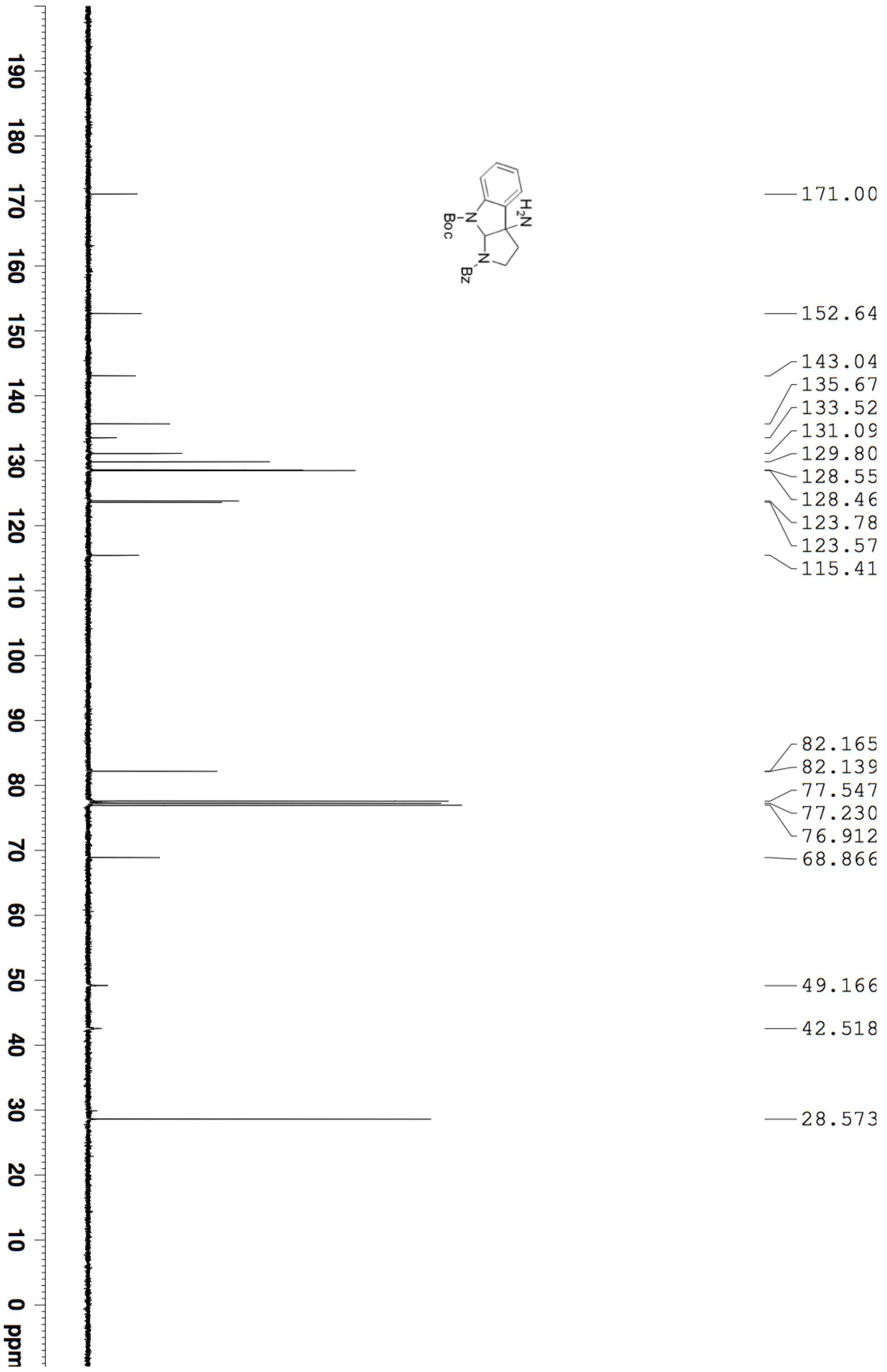
171.00
152.64
143.04
135.67
133.52
131.09
129.80
128.55
128.46
123.78
123.57
115.41
82.165
82.139
77.547
77.230
76.912
68.866
49.166
42.518
28.573
H2N
N
Boc
N
Bz
190 180 170 160 150 140 130 120 110 100 90 80 70 60 50 40 30 20 10 0 ppm

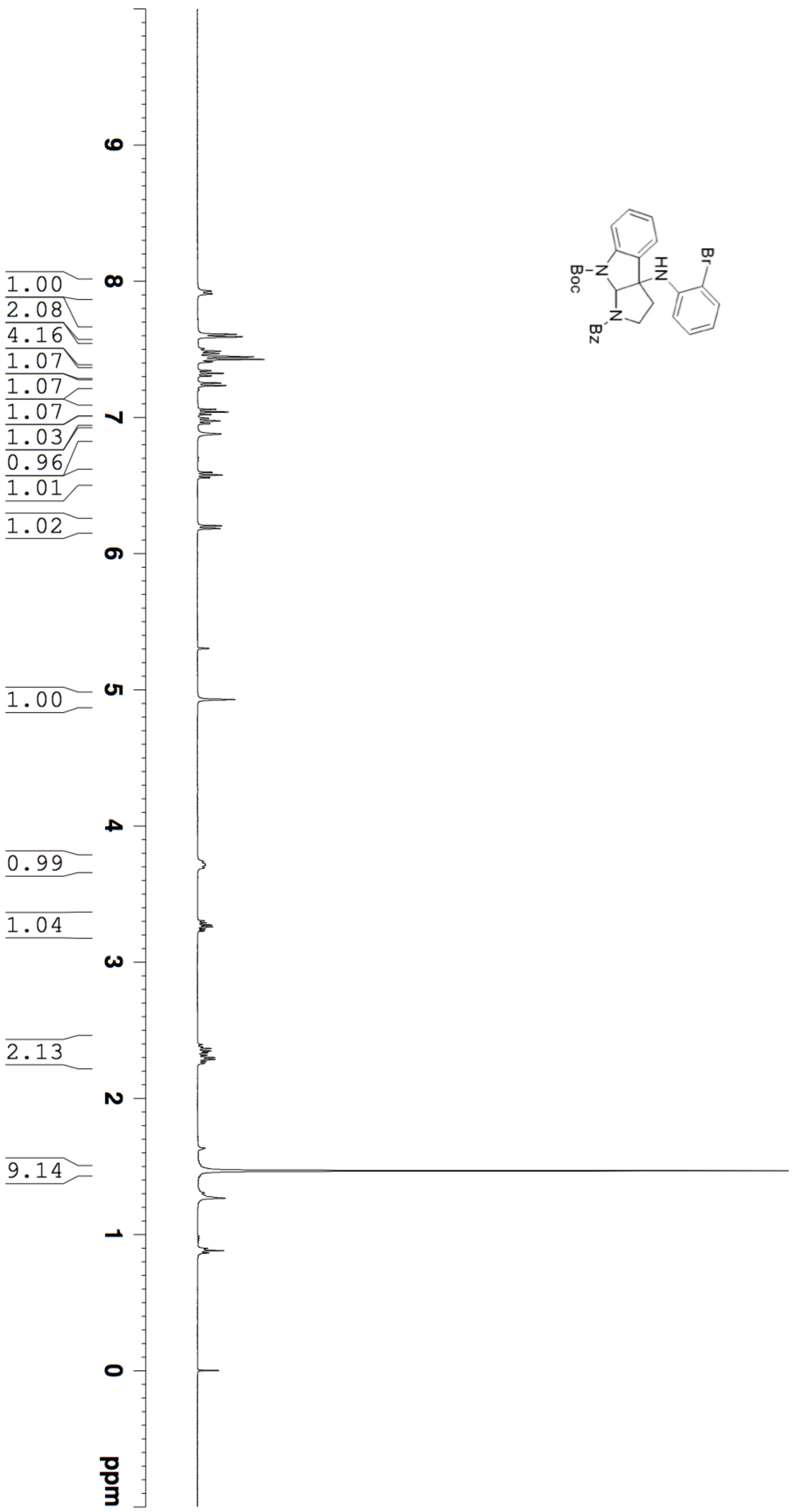

ppm
9
8
7
6
5
4
3
2
1
0
1.00
2.08
4.16
1.07
1.07
1.07
1.03
0.96
1.01
1.02
1.00
0.99
1.04
2.13
9.14
HN
Br
N
Boc
Bz

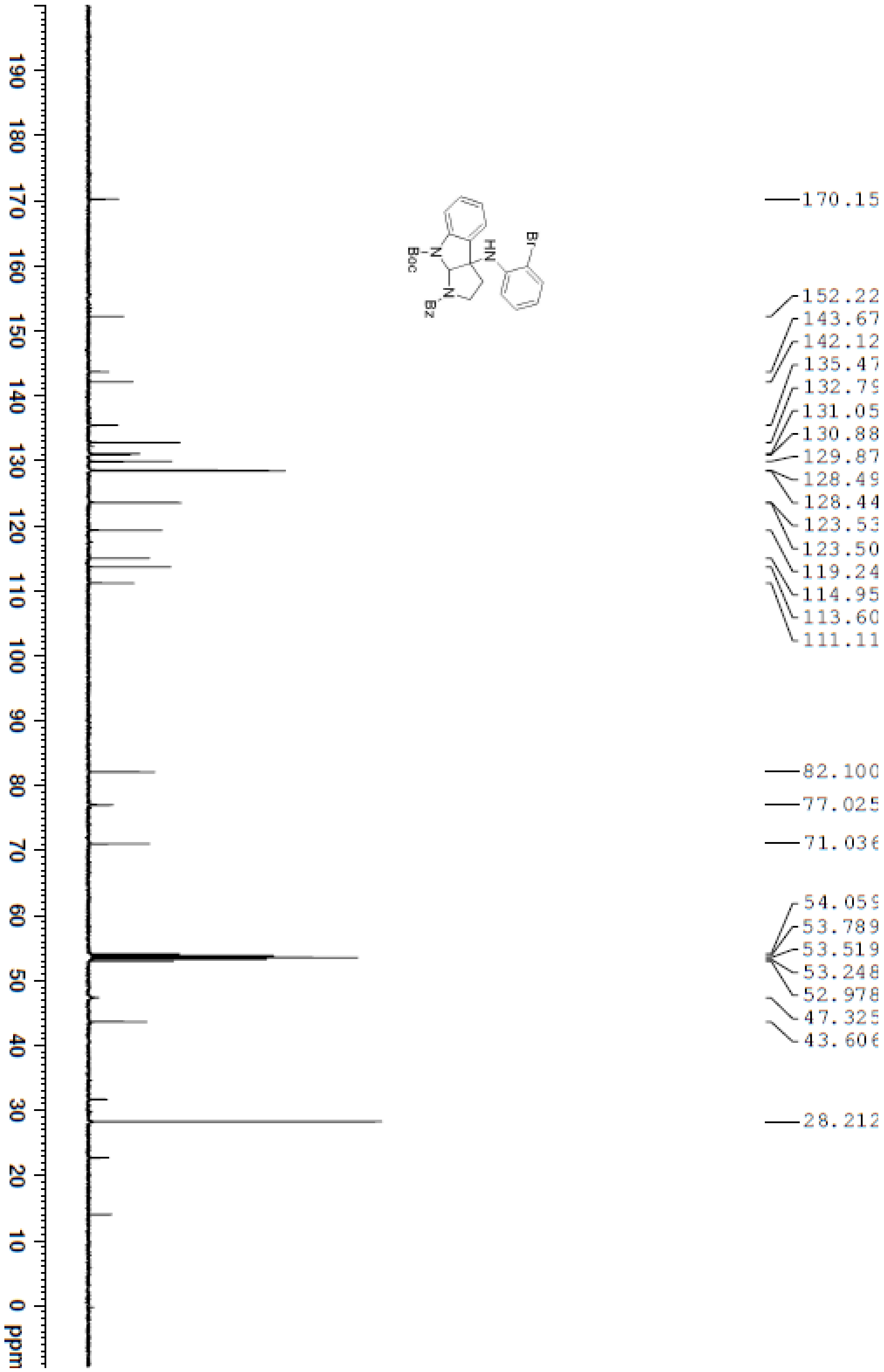
Br
HN
N
Boc
N
Bz
170.15
152.22
143.67
142.12
135.47
132.79
131.05
130.88
129.87
128.49
128.44
123.53
123.50
119.24
114.95
113.60
111.11
82.100
77.025
71.036
54.059
53.789
53.519
53.248
52.978
47.325
43.606
28.212
ppm

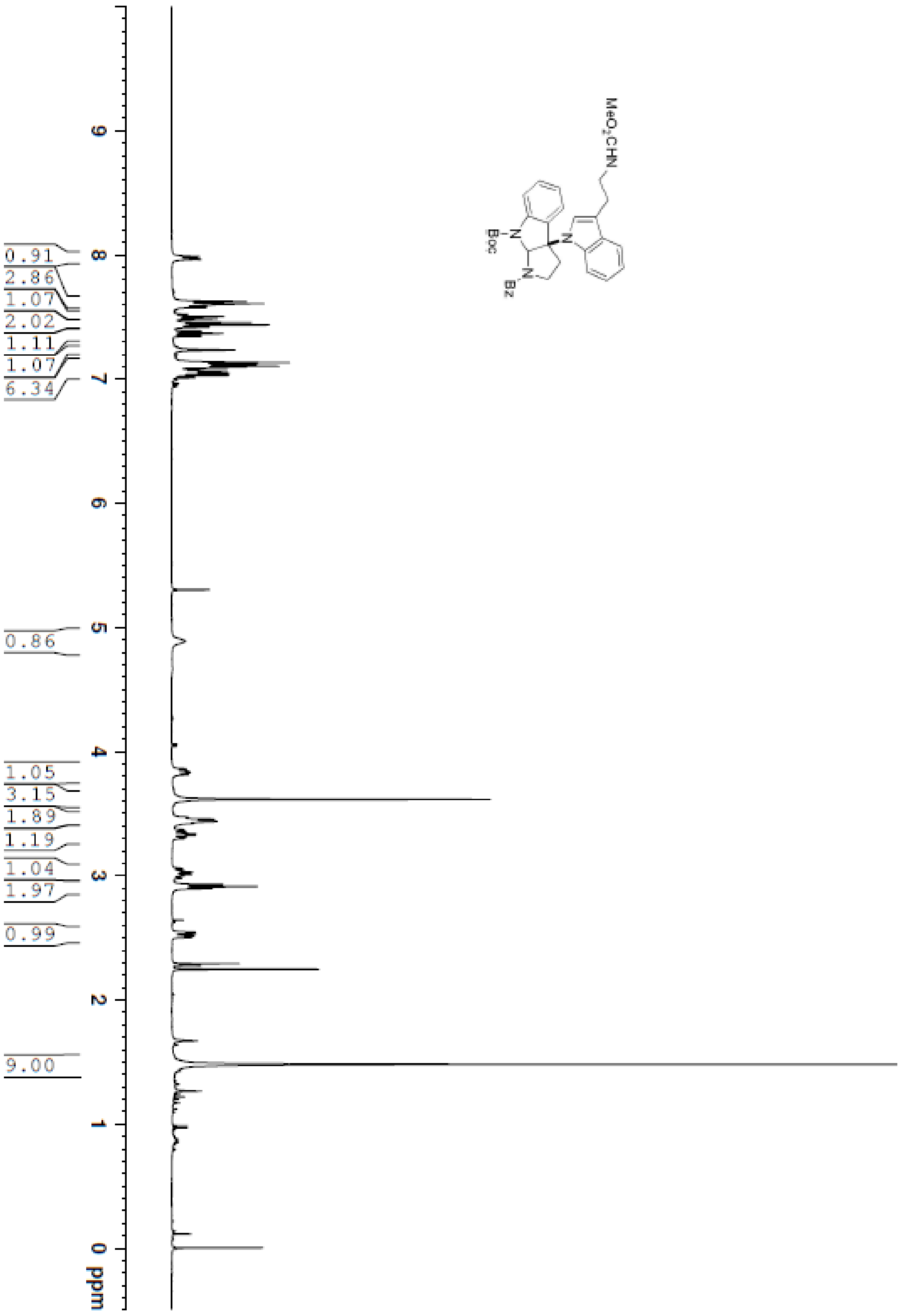
MeO2CHN
N
N
N
Boc
Bz
0.91
2.86
1.07
2.02
1.11
1.07
6.34
0.86
1.05
3.15
1.89
1.19
1.04
1.97
0.99
9.00
9
8
7
6
5
4
3
2
1
0 ppm

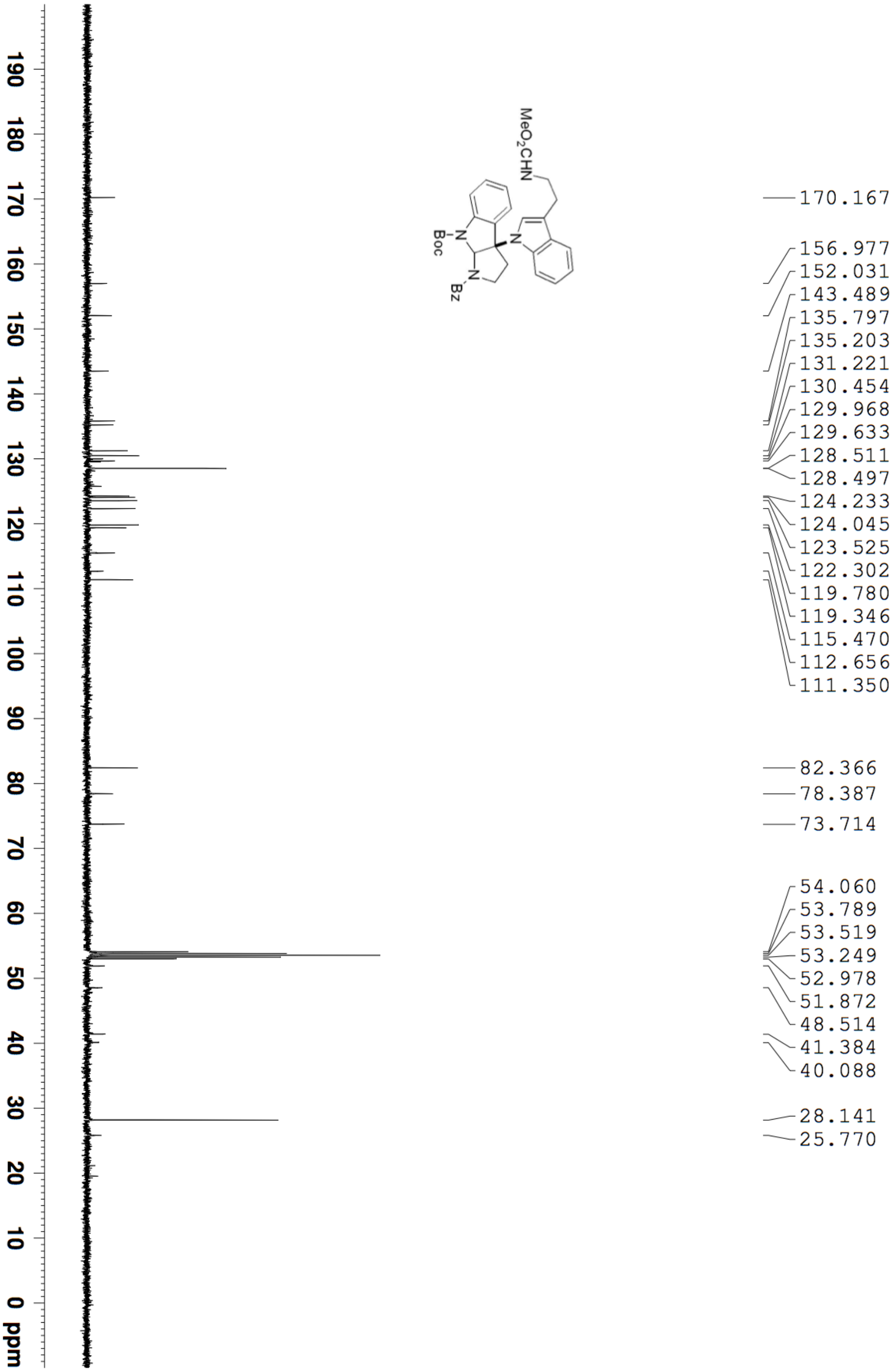
170.167
156.977
152.031
143.489
135.797
135.203
131.221
130.454
129.968
129.633
128.511
128.497
124.233
124.045
123.525
122.302
119.780
119.346
115.470
112.656
111.350
82.366
78.387
73.714
54.060
53.789
53.519
53.249
52.978
51.872
48.514
41.384
40.088
28.141
25.770
MeO2CHN
N
Boc
Bz
ppm

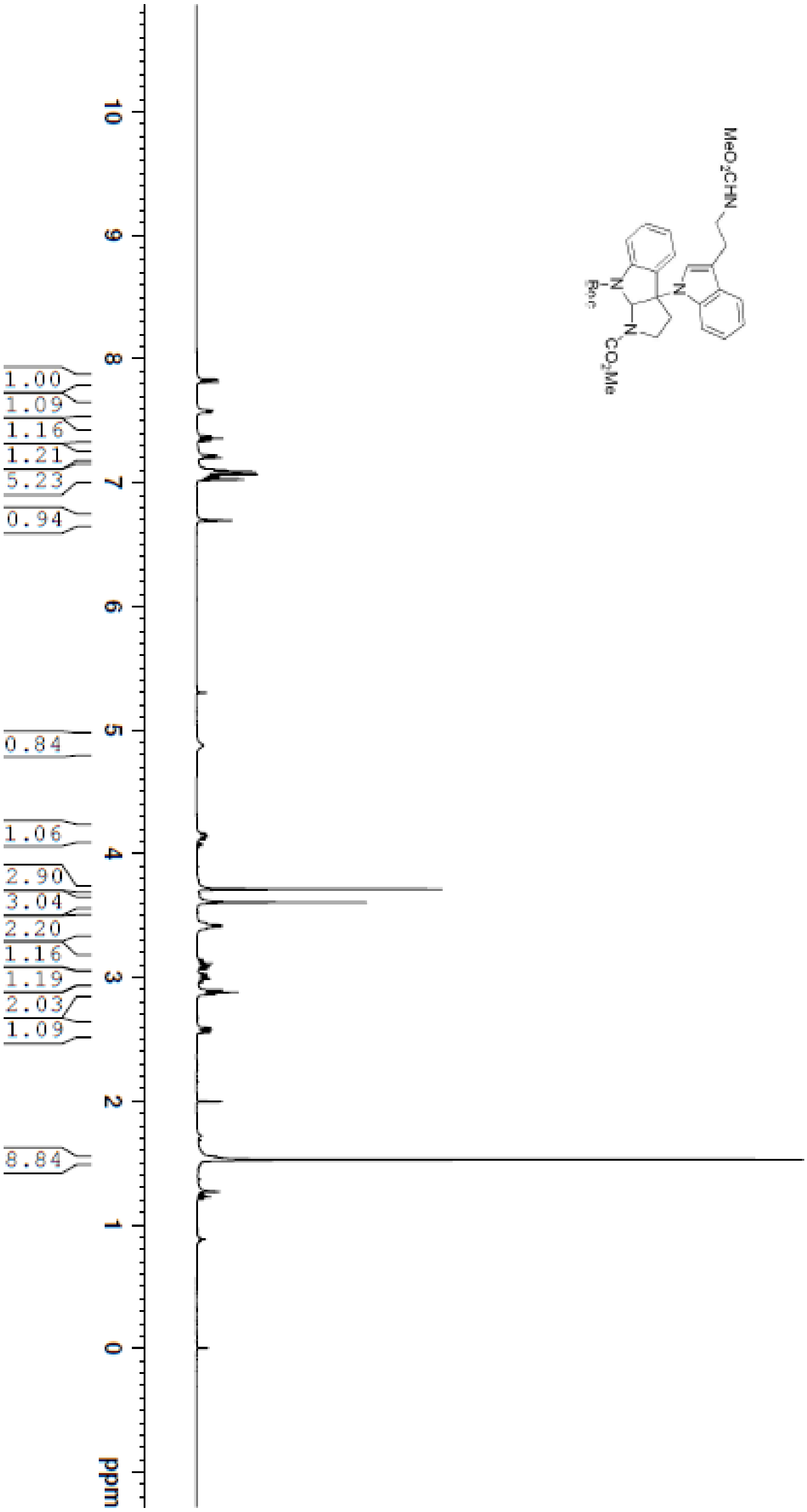
MeO2CHN
N
N
Boc
N
CO2Me
1.00
1.09
1.16
1.21
5.23
0.94
0.84
1.06
2.90
3.04
2.20
1.16
1.19
2.03
1.09
8.84
10
9
8
7
6
5
4
3
2
1
0
ppm

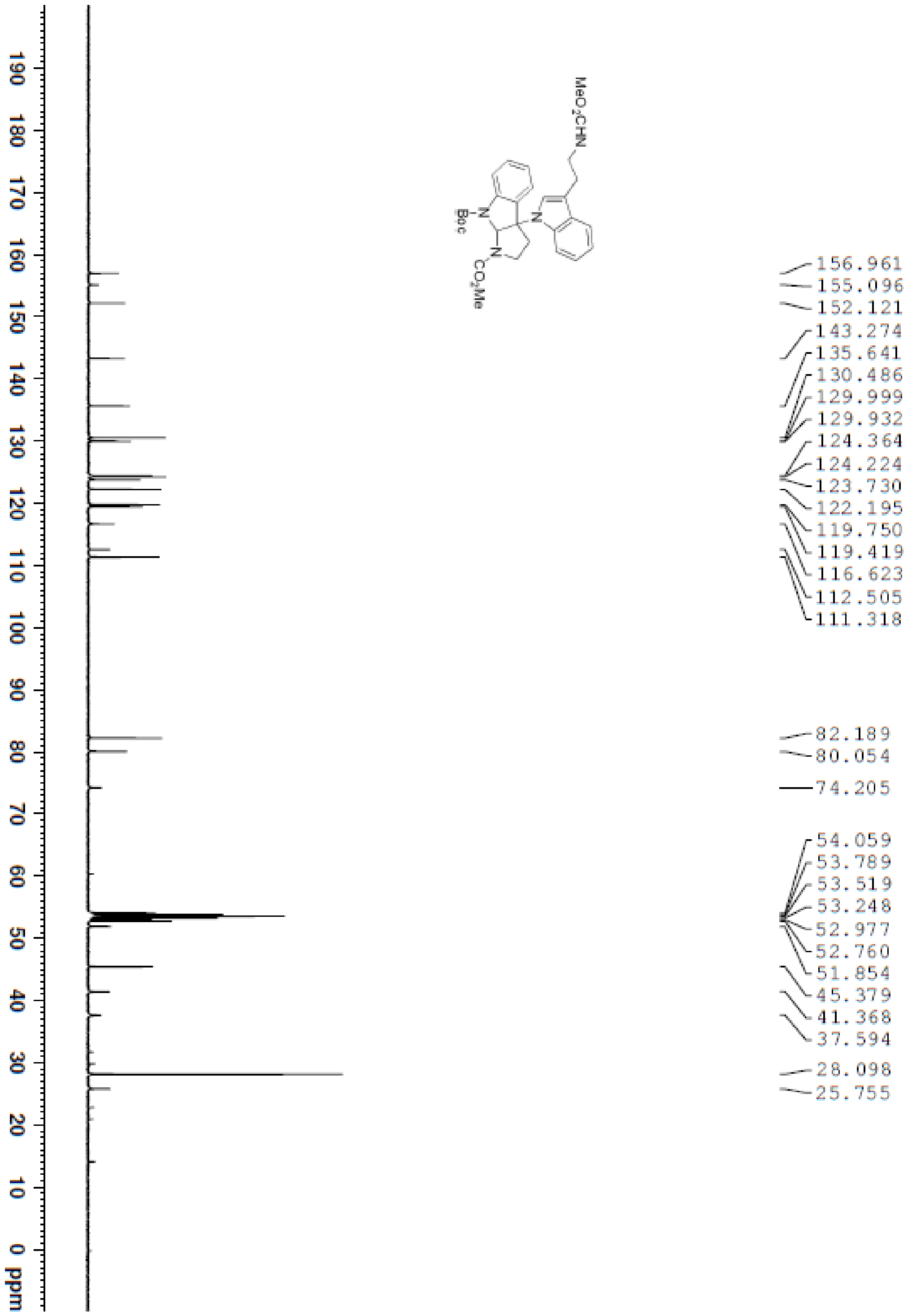
MeO2CHN
N
N
N
Boc
CO2Me
156.961
155.096
152.121
143.274
135.641
130.486
129.999
129.932
124.364
124.224
123.730
122.195
119.750
119.419
116.623
112.505
111.318
82.189
80.054
74.205
54.059
53.789
53.519
53.248
52.977
52.760
51.854
45.379
41.368
37.594
28.098
25.755
190 180 170 160 150 140 130 120 110 100 90 80 70 60 50 40 30 20 10 0 ppm

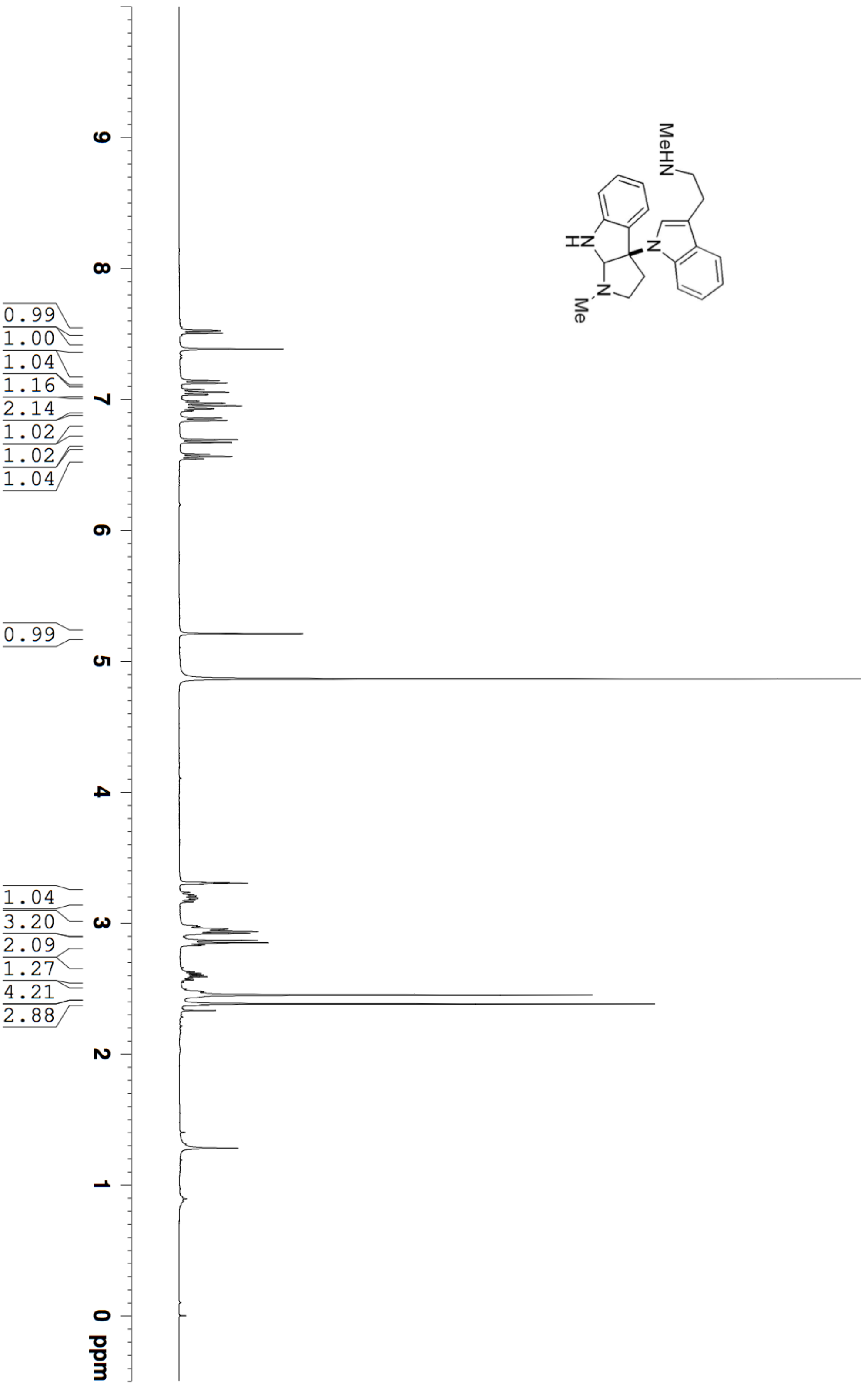
MeHN
N
H
N
N
Me
0.99
1.00
1.04
1.16
2.14
1.02
1.02
1.04
0.99
1.04
3.20
2.09
1.27
4.21
2.88
9
8
7
6
5
4
3
2
1
0 ppm

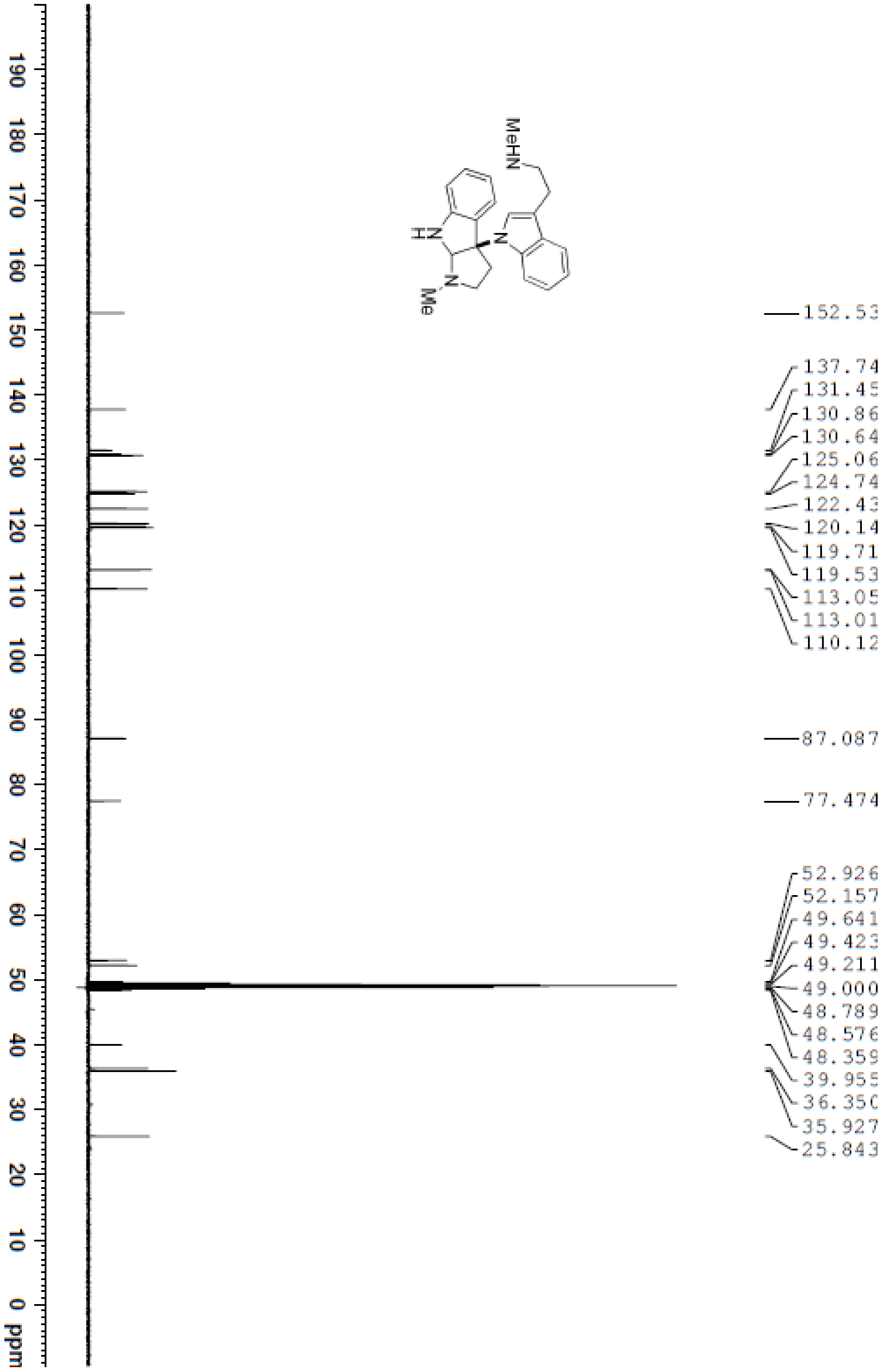
152.53
137.74
131.45
130.86
130.64
125.06
124.74
122.43
120.14
119.71
119.53
113.05
113.01
110.12
87.087
77.474
52.926
52.157
49.641
49.423
49.211
49.000
48.789
48.576
48.359
39.955
36.350
35.927
25.843
MeHN
N
N
H
N
Me
ppm

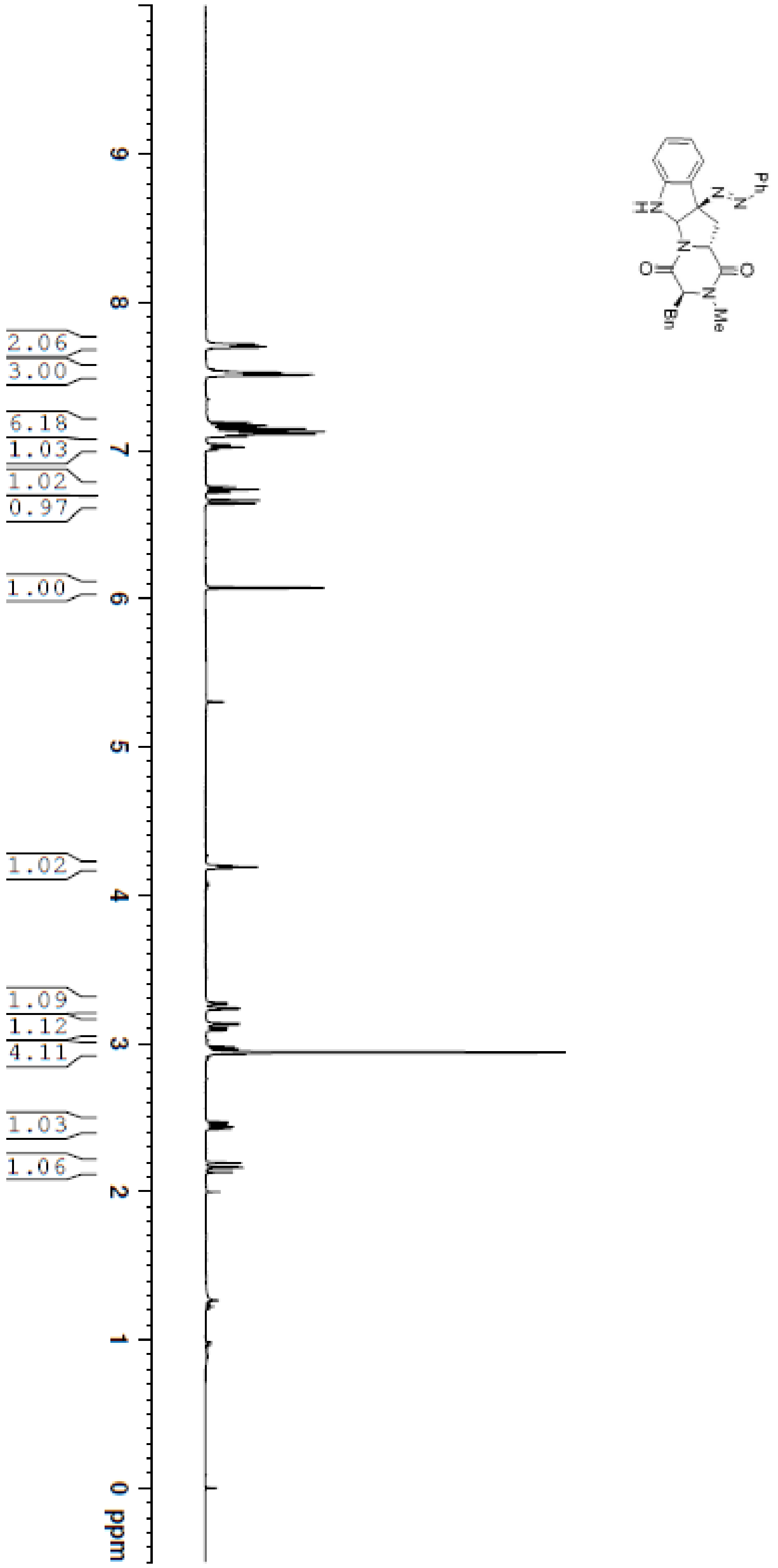

2.06
3.00
6.18
1.03
1.02
0.97
1.00
1.02
1.09
1.12
4.11
1.03
1.06
9
8
7
6
5
4
3
2
1
0 ppm
Ph
N
HN
O
Bn
Me

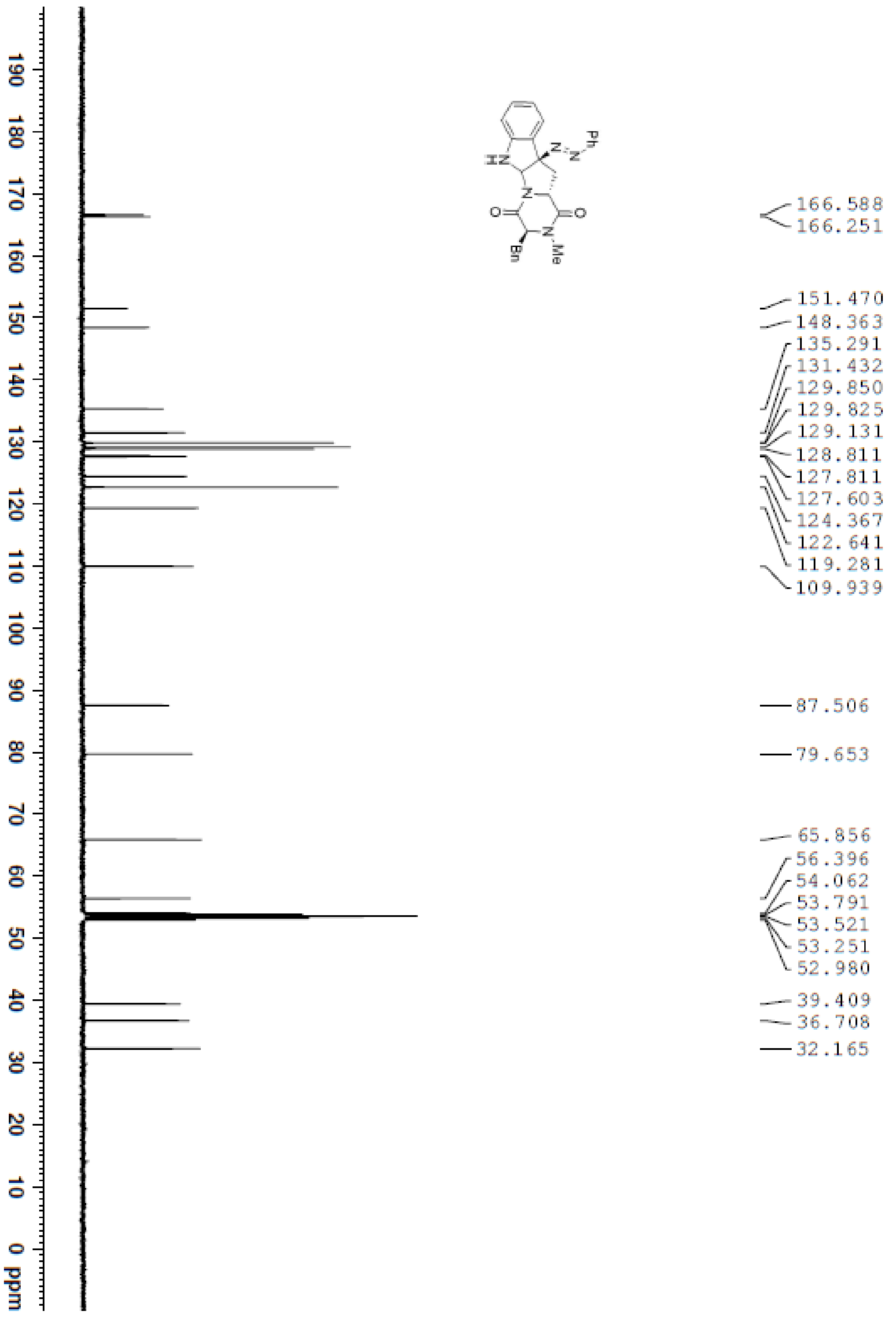
166.588
166.251
151.470
148.363
135.291
131.432
129.850
129.825
129.131
128.811
127.811
127.603
124.367
122.641
119.281
109.939
87.506
79.653
65.856
56.396
54.062
53.791
53.521
53.251
52.980
39.409
36.708
32.165

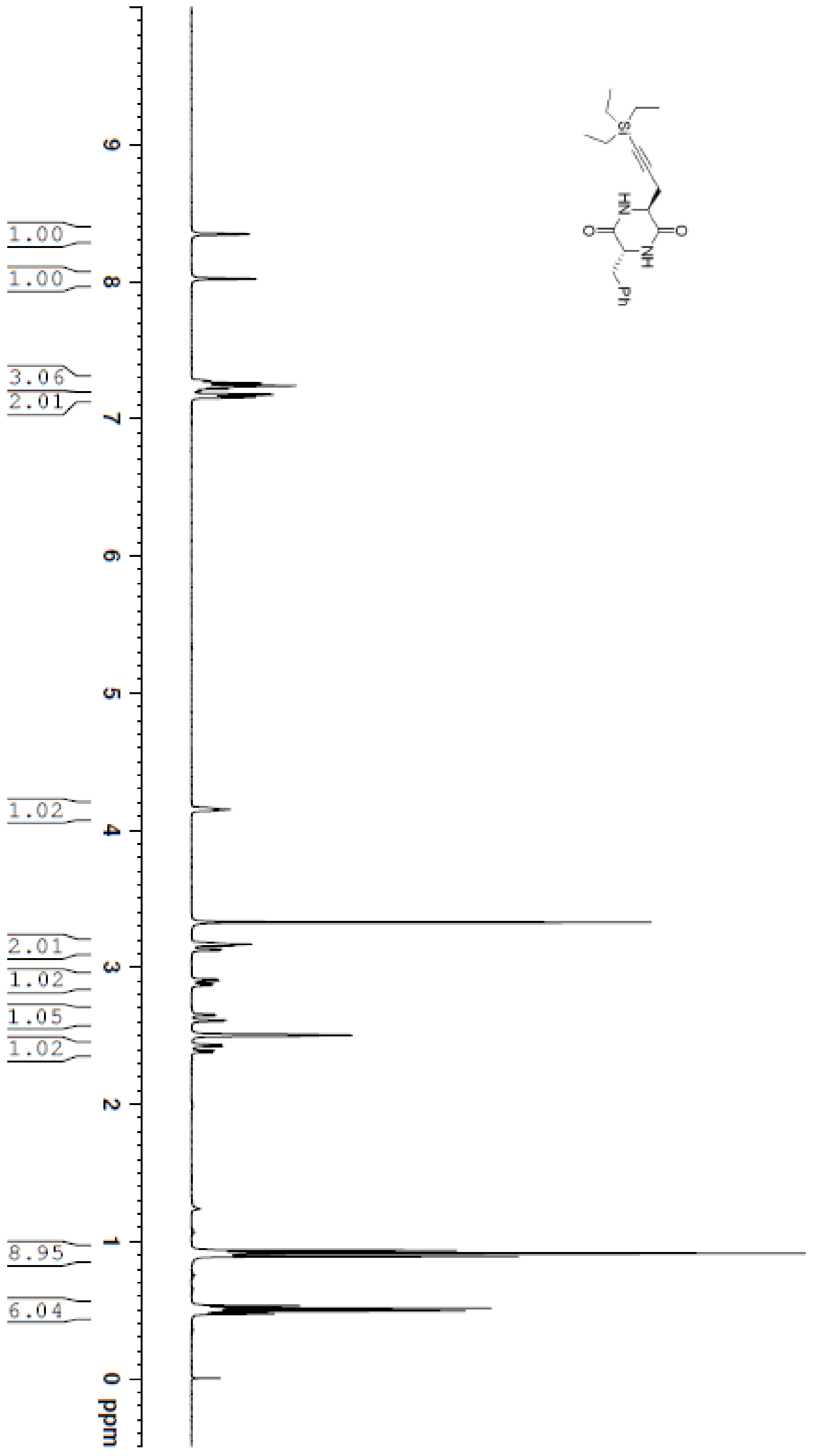

9
8
7
6
5
4
3
2
1
0 ppm
1.00
1.00
3.06
2.01
1.02
2.01
1.02
1.05
1.02
8.95
6.04

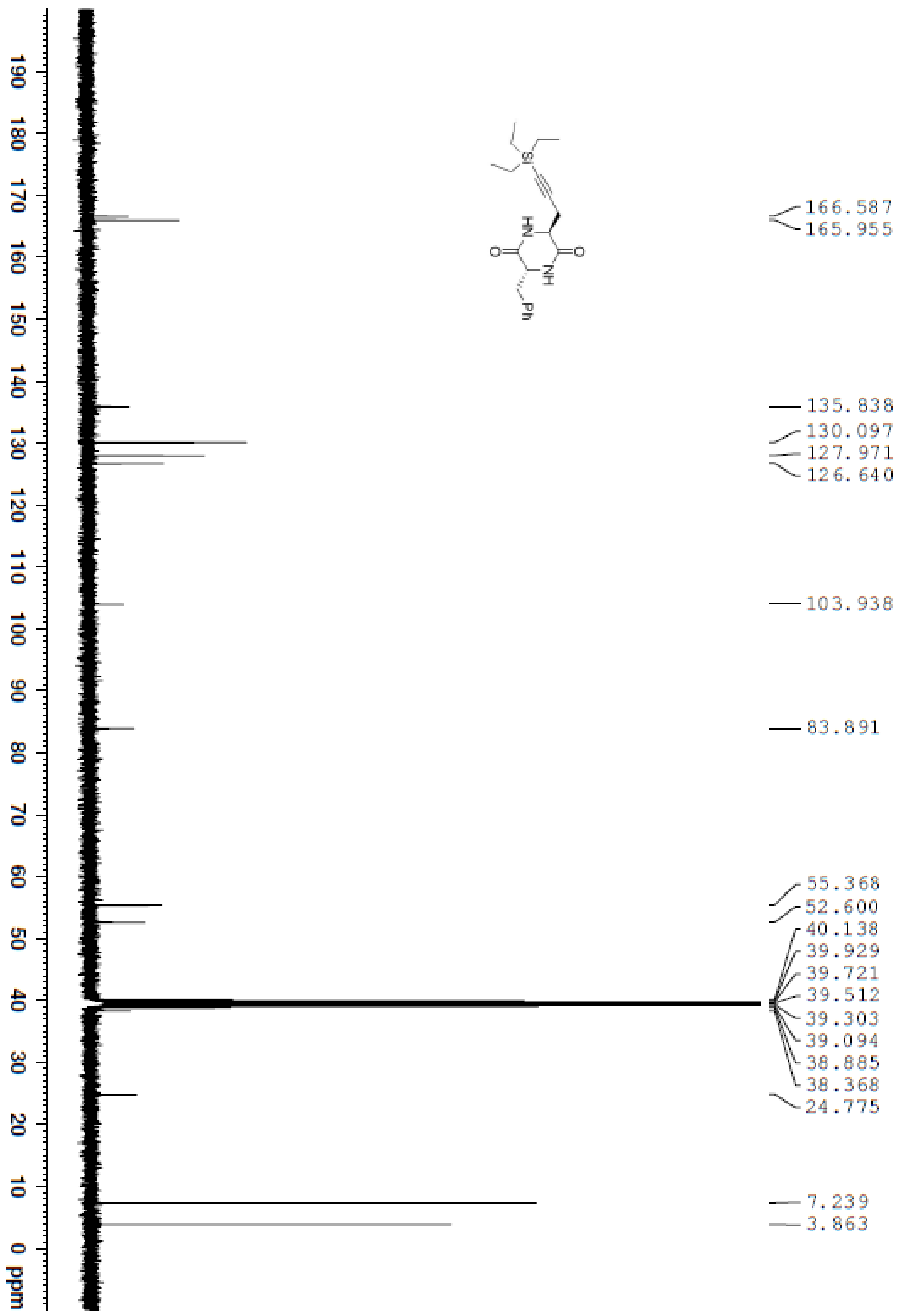

166.587
165.955
135.838
130.097
127.971
126.640
103.938
83.891
55.368
52.600
40.138
39.929
39.721
39.512
39.303
39.094
38.885
38.368
24.775
7.239
3.863
ppm

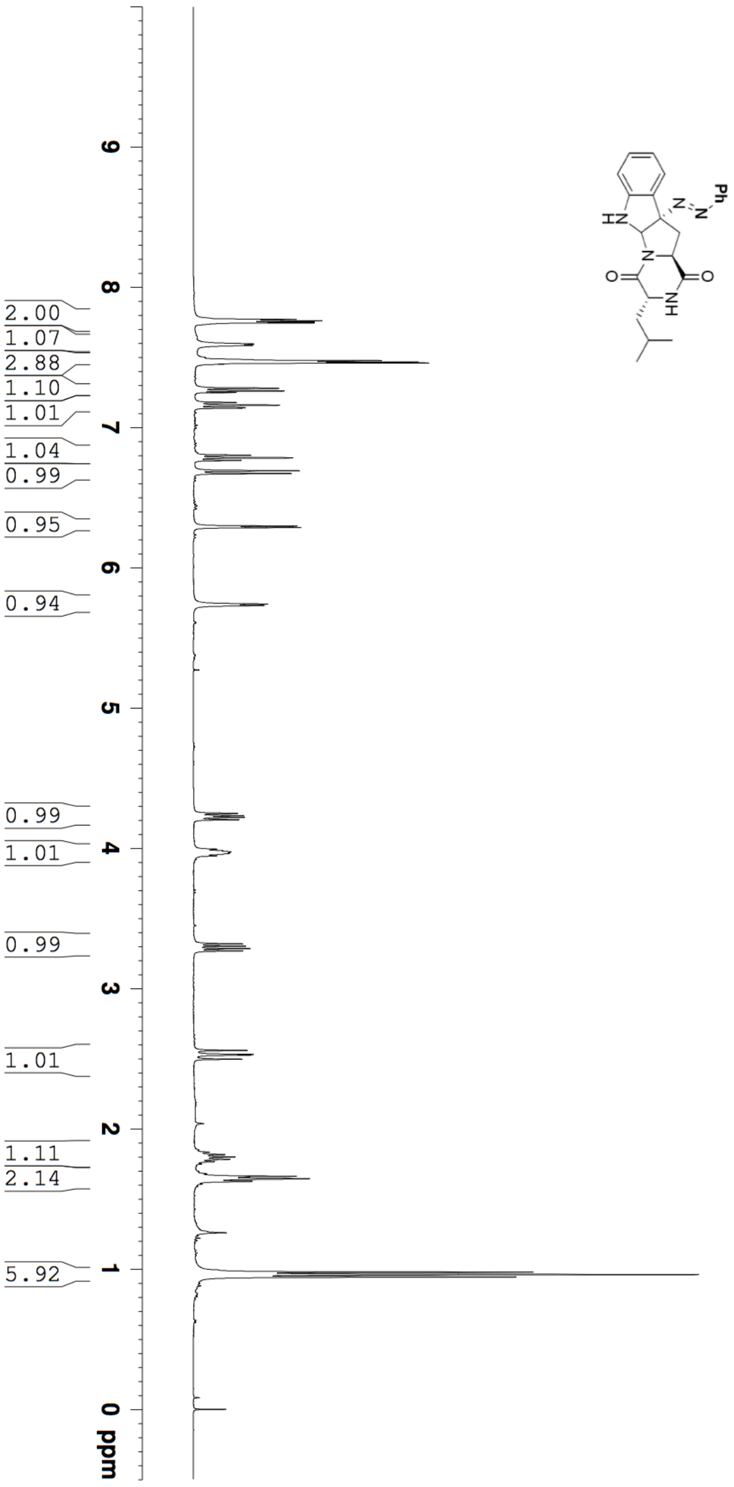
2.00
1.07
2.88
1.10
1.01
1.04
0.99
0.95
0.94
0.99
1.01
0.99
1.01
1.11
2.14
5.92
9
8
7
6
5
4
3
2
1
0
ppm
HN
N
O
O
NH
N
N
Ph

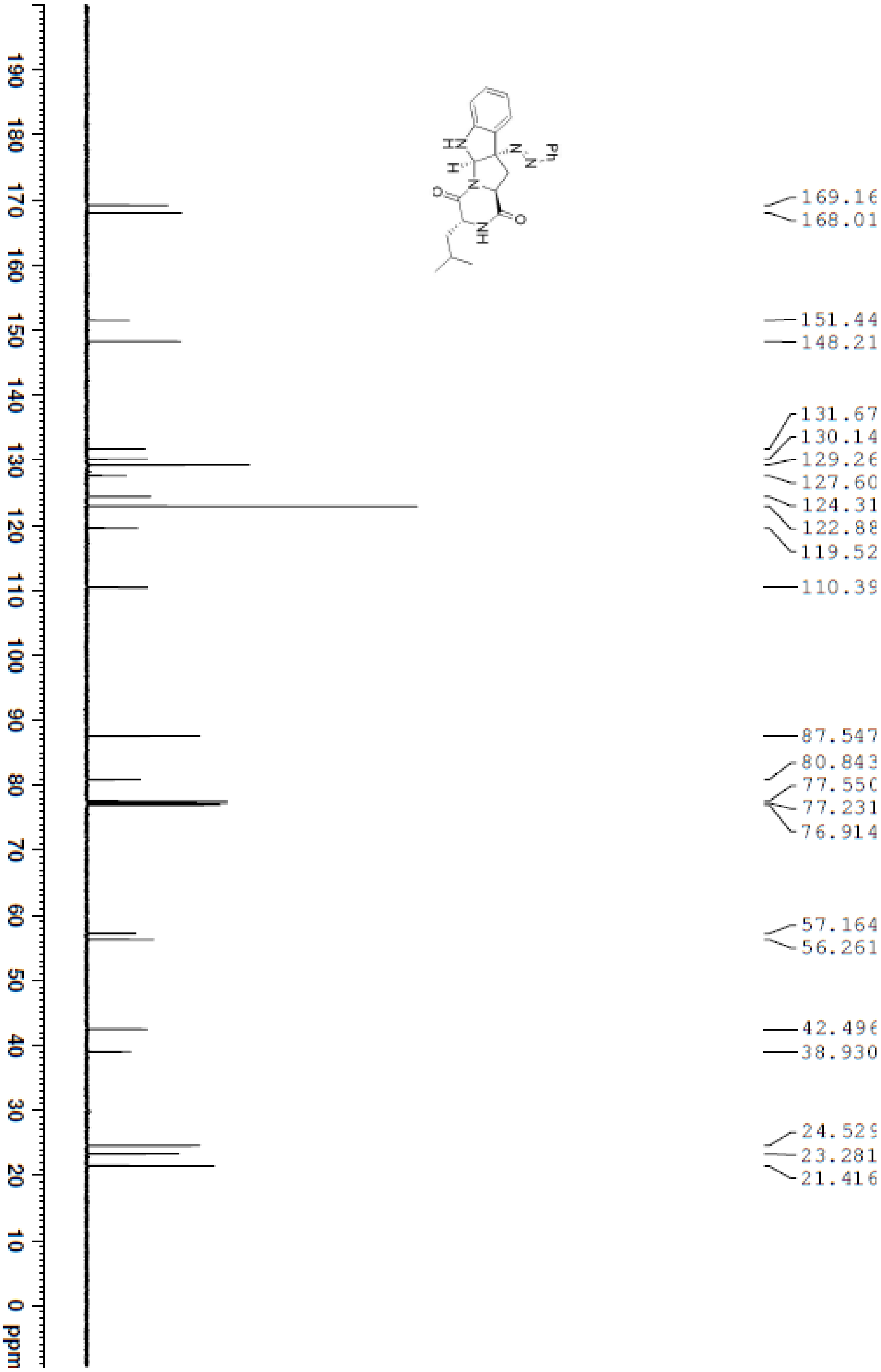
169.16
168.01
151.44
148.21
131.67
130.14
129.26
127.60
124.31
122.88
119.52
110.39
87.547
80.843
77.550
77.231
76.914
57.164
56.261
42.496
38.930
24.529
23.281
21.416
ppm

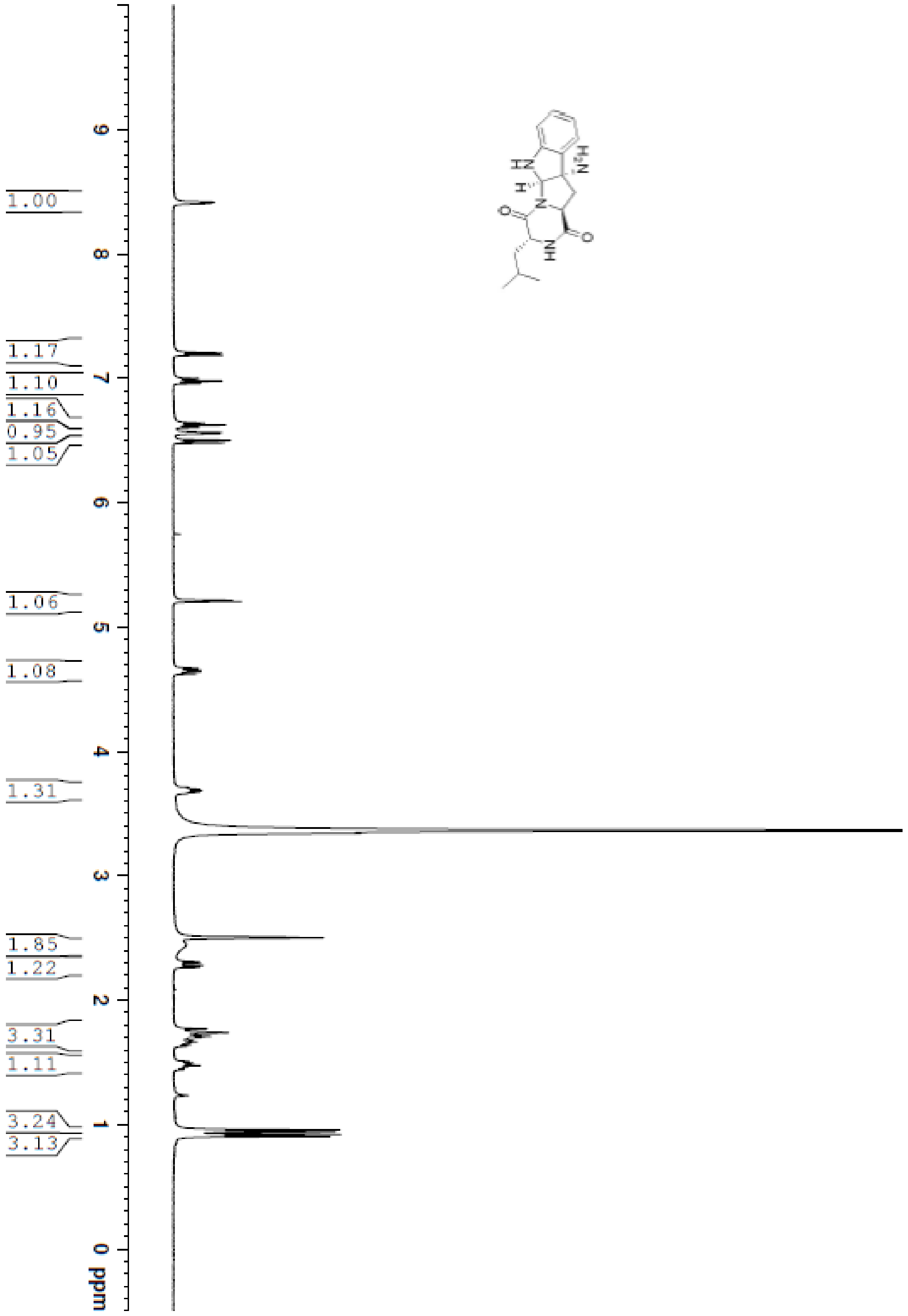
1.00
1.17
1.10
1.16
0.95
1.05
1.06
1.08
1.31
1.85
1.22
3.31
1.11
3.24
3.13
9
8
7
6
5
4
3
2
1
0 ppm
H2N
N
H
H
N
O
NH
O

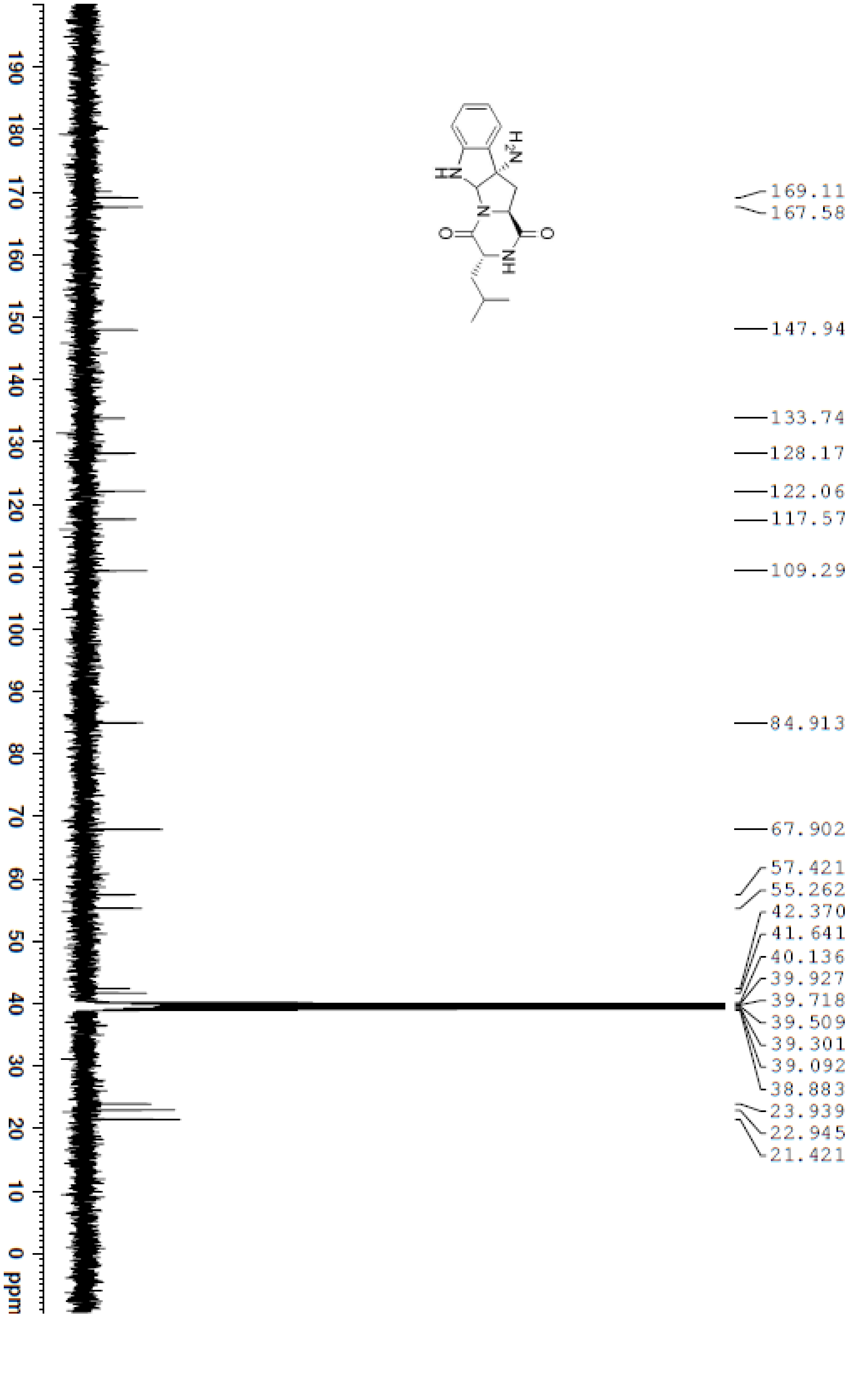
169.11
167.58
147.94
133.74
128.17
122.06
117.57
109.29
84.913
67.902
57.421
55.262
42.370
41.641
40.136
39.927
39.718
39.509
39.301
39.092
38.883
23.939
22.945
21.421

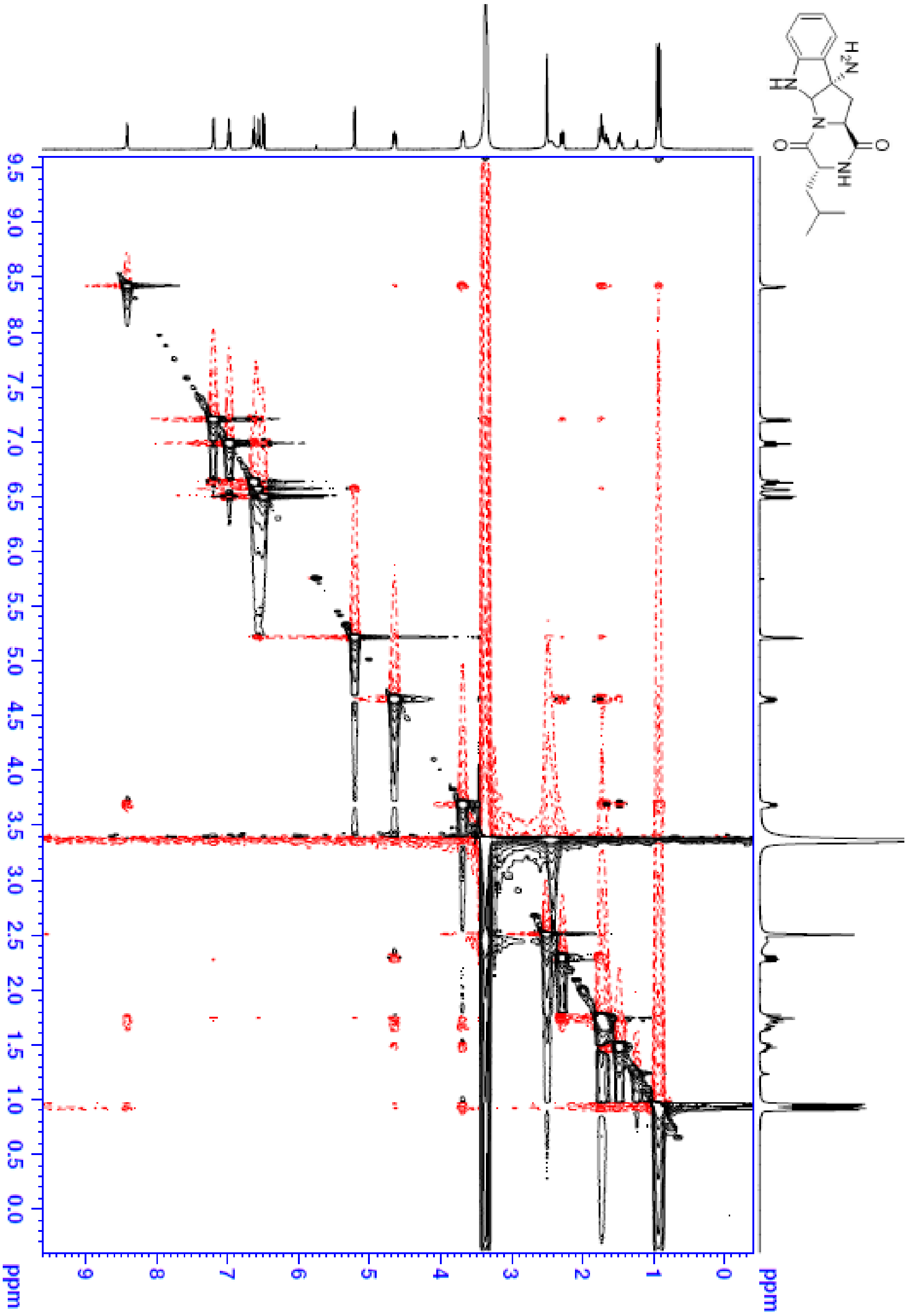

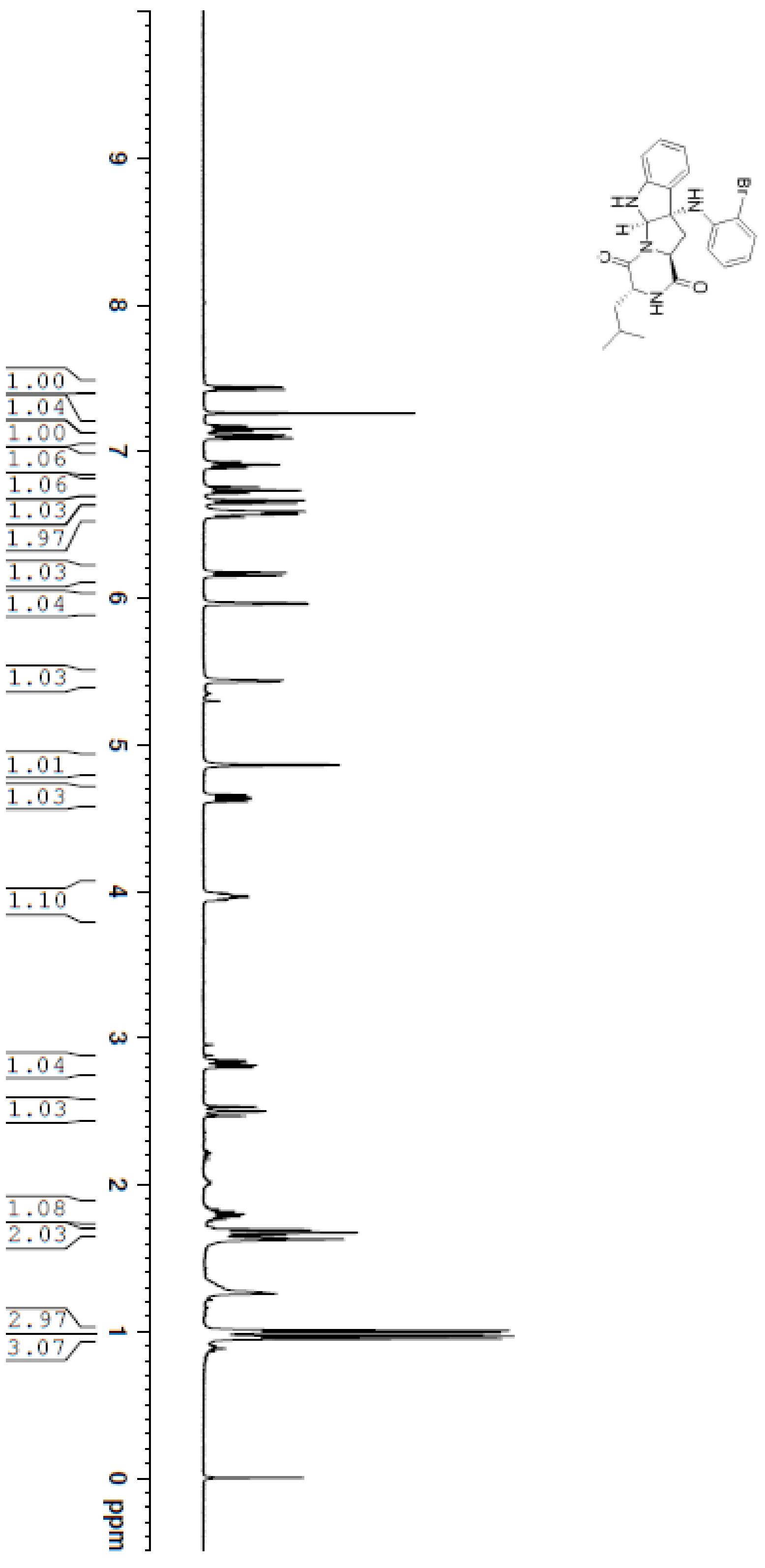

1.00
1.04
1.00
1.06
1.06
1.03
1.97
1.03
1.04
1.03
1.01
1.03
1.10
1.04
1.03
1.08
2.03
2.97
3.07
9
8
7
6
5
4
3
2
1
0 ppm
Br
HN
NH
O

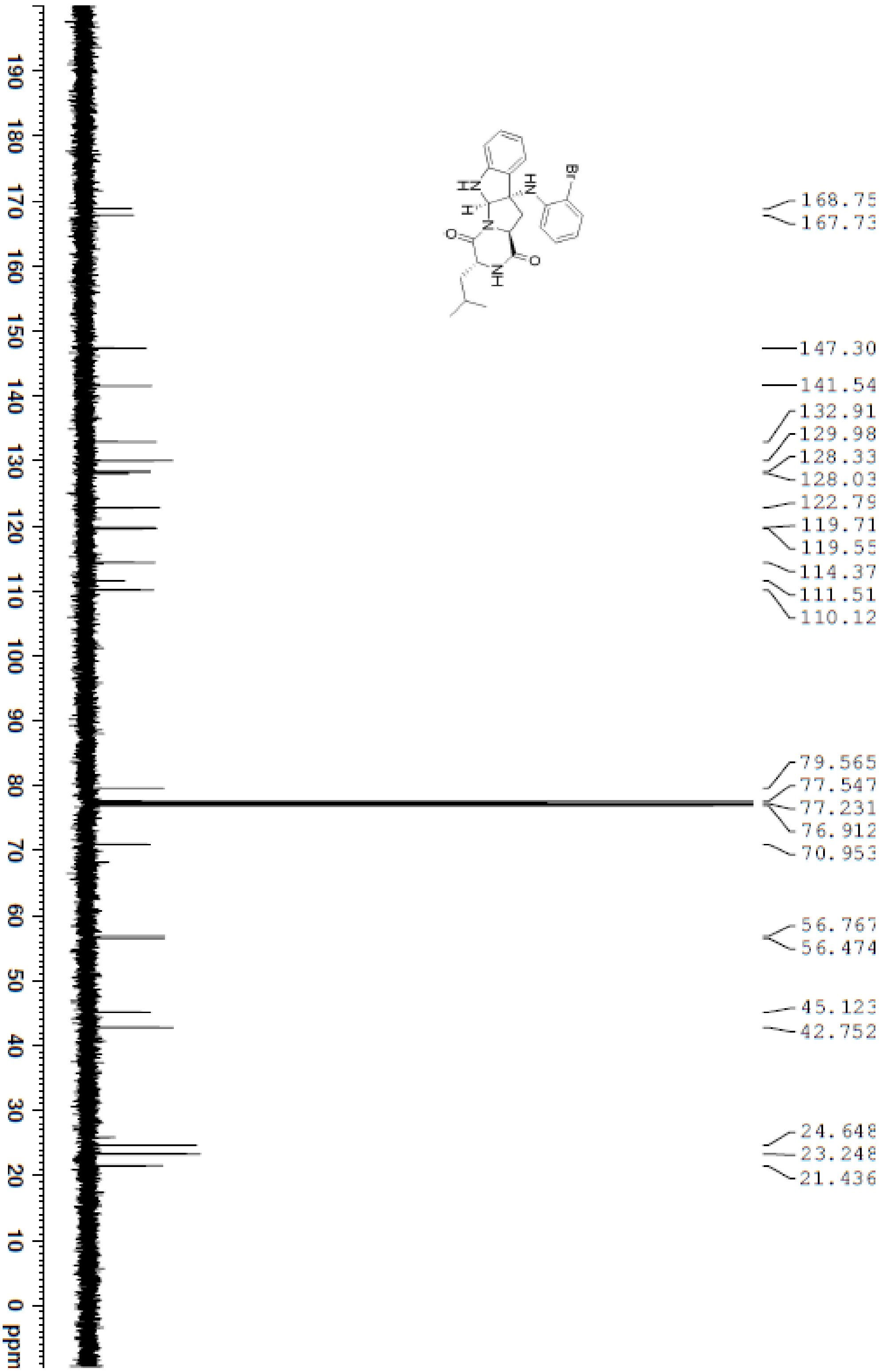
168.75
167.73
147.30
141.54
132.91
129.98
128.33
128.03
122.79
119.71
119.55
114.37
111.51
110.12
79.565
77.547
77.231
76.912
70.953
56.767
56.474
45.123
42.752
24.648
23.248
21.436
ppm

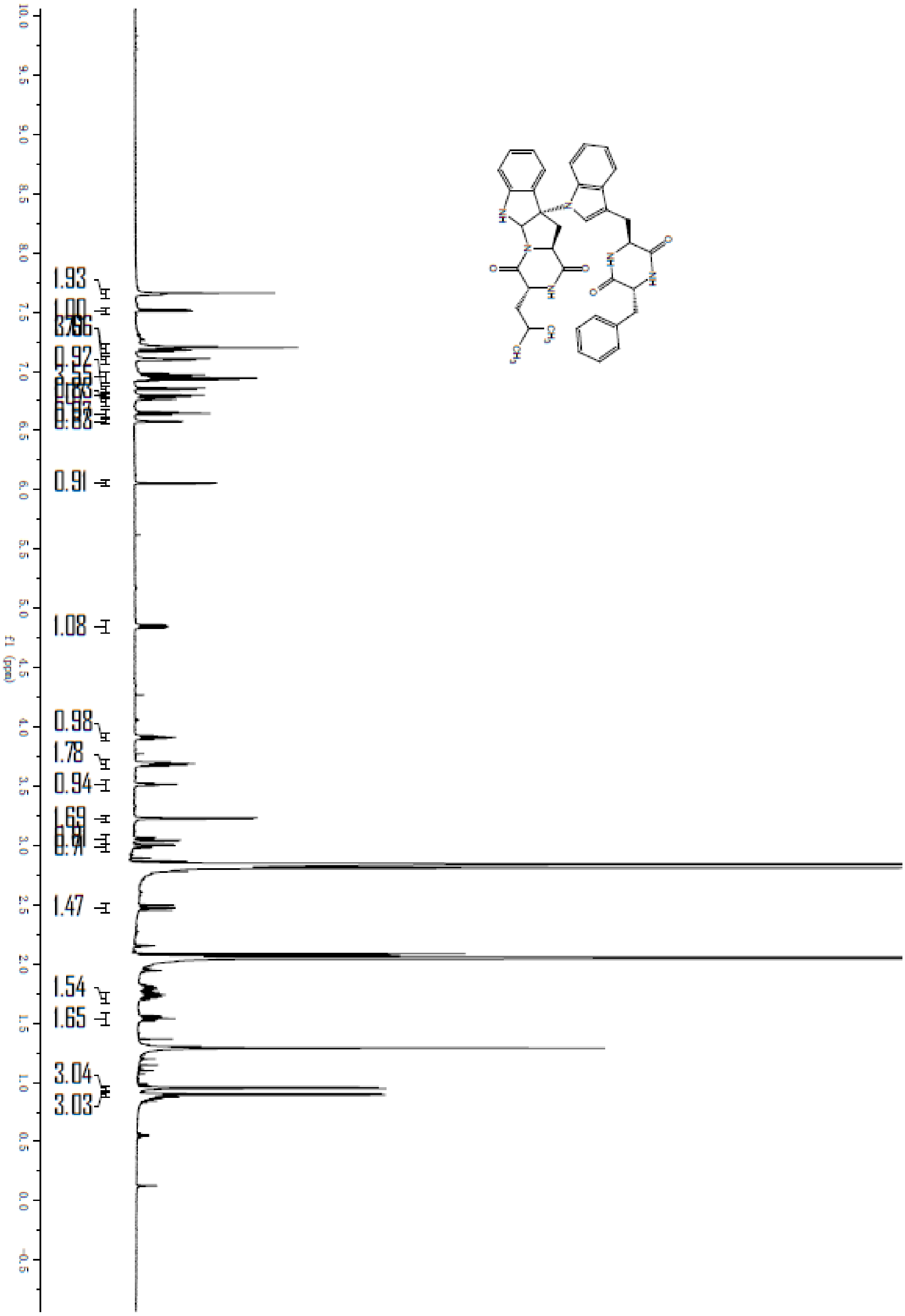

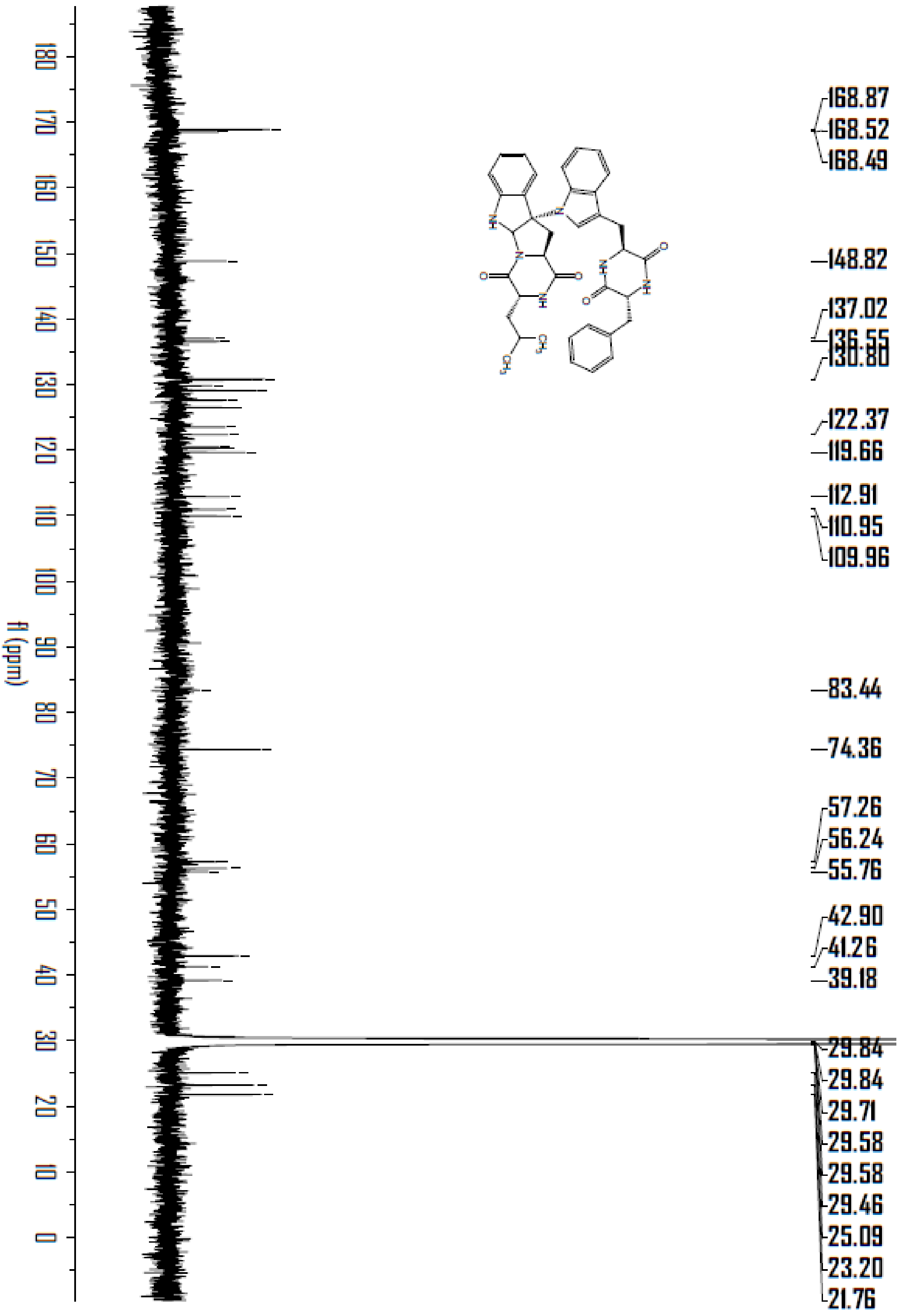
168.87
168.52
168.49
148.82
137.02
136.55
130.80
122.37
119.66
112.91
110.95
109.96
83.44
74.36
57.26
56.24
55.76
42.90
41.26
39.18
29.84
29.84
29.71
29.58
29.58
29.46
25.09
23.20
21.76
f1 (ppm)
